# Naringenin restricts the colonization and growth of *Ralstonia solanacearum* in tobacco mutant KCB-1

**DOI:** 10.1093/plphys/kiae185

**Published:** 2024-04-04

**Authors:** Haoqi Shi, Jiale Jiang, Wen Yu, Yazhi Cheng, Shengxin Wu, Hao Zong, Xiaoqiang Wang, Anming Ding, Weifeng Wang, Yuhe Sun

**Affiliations:** Key Laboratory for Tobacco Gene Resources, Tobacco Research Institute of Chinese Academy of Agricultural Sciences, Qingdao 266101, China; Graduate School of Chinese Academy of Agricultural Sciences, Beijing 100081, China; Key Laboratory for Tobacco Gene Resources, Tobacco Research Institute of Chinese Academy of Agricultural Sciences, Qingdao 266101, China; Graduate School of Chinese Academy of Agricultural Sciences, Beijing 100081, China; Fujian Institute of Tobacco Agricultural Sciences, Fuzhou 350003, China; Fujian Institute of Tobacco Agricultural Sciences, Fuzhou 350003, China; Fujian Institute of Tobacco Agricultural Sciences, Fuzhou 350003, China; Shandong Linyi Tobacco Co., Ltd., Linyi 276000, China; Tobacco Research Institute of Chinese Academy of Agricultural Sciences, Qingdao 266101, China; Key Laboratory for Tobacco Gene Resources, Tobacco Research Institute of Chinese Academy of Agricultural Sciences, Qingdao 266101, China; Key Laboratory for Tobacco Gene Resources, Tobacco Research Institute of Chinese Academy of Agricultural Sciences, Qingdao 266101, China; Key Laboratory for Tobacco Gene Resources, Tobacco Research Institute of Chinese Academy of Agricultural Sciences, Qingdao 266101, China

## Abstract

Bacterial wilt severely jeopardizes plant growth and causes enormous economic loss in the production of many crops, including tobacco (*Nicotiana tabacum*). Here, we first demonstrated that the roots of bacterial wilt-resistant tobacco mutant KCB-1 can limit the growth and reproduction of *Ralstonia solanacearum*. Secondly, we demonstrated that KCB-1 specifically induced an upregulation of naringenin content in root metabolites and root secretions. Further experiments showed that naringenin can disrupt the structure of *R*. *solanacearum*, inhibit the growth and reproduction of *R. solanacearum*, and exert a controlling effect on bacterial wilt. Exogenous naringenin application activated the resistance response in tobacco by inducing the burst of reactive oxygen species and salicylic acid deposition, leading to transcriptional reprogramming in tobacco roots. Additionally, both external application of naringenin in CB-1 and overexpression of the *Nicotiana tabacum chalcone isomerase* (*NtCHI*) gene, which regulates naringenin biosynthesis, in CB-1 resulted in a higher complexity of their inter-root bacterial communities than in untreated CB-1. Further analysis showed that naringenin could be used as a marker for resistant tobacco. The present study provides a reference for analyzing the resistance mechanism of bacterial wilt-resistant tobacco and controlling tobacco bacterial wilt.

## Teaser

Bacterial wilt-resistant mutant KCB-1 restricts colonization and growth of *Ralstonia solanacearum* by specifically inducing increased naringenin content.

## Introduction

*Ralstonia solanacearum* (*R. solanacearum*) is a soilborne pathogen that affects a large number of plants and causes huge economic losses to world crops (Shi et al. 2022a, 2022b). *R*. *solanacearum* enter the xylem conduit through the injury of the plant root system and later move to the aboveground. Under high temperature and high humidity conditions, *R. solanacearum* proliferates and causes blockage of the xylem ducts, which leads to water loss and wilting of plant leaves, resulting in the emergence of bacterial wilt (Shi et al. 2023). Currently, the control of bacterial wilt is mainly through pesticides, microbial fungicides, etc., but the most green and effective way is through resistant varieties (Shi et al. 2022a, 2022b). Tobacco (*Nicotiana tabacum*), as an important cash crop, is also widely affected by bacterial wilt. Currently, tobacco grown in China is dominated by varieties that are susceptible or moderately resistant to bacterial wilt, which is extremely detrimental to safeguarding the economic benefits of tobacco. Improving susceptible tobacco varieties through near-isogenic lines (NIL) generated by ethyl methanesulfonate (EMS) mutagenesis is a solution to maximize the quality and yield of tobacco itself. However, there is a lack of knowledge in analyzing the resistance mechanisms of these bacterial wilt-resistant tobaccos.

*R. solanacearum* have a large distribution range and many variants, and there are still no primary genes localized in plants that can control all *R. solanacearum* variants. Therefore, researchers have attempted to resolve plant resistance mechanisms through the plant's own physical and chemical defenses (Kashyap et al. 2021). Studies have shown that infestation formation was observed at infected sites in both bacterial wilt-resistant tomato and potato (Grimault et al. 1994; Ferreira et al. 2017), whereas infestation formation was delayed and less centralized in bacterial wilt-susceptible tomato (Grimault et al. 1994). Many xylem conduits that were not colonized by *R. solanacearum* were also clogged with infiltrants, but pathogen multiplication was not restricted (Grimault et al. 1994). Formation of infiltrative bodies was not observed in tomato varieties susceptible to *R. solanacearum* when not inoculated with *R. solanacearum*, whereas they were present in resistant tomatoes. This preformed physical defense was more timely and effective in the face of *R. solanacearum* infestation.

For chemical defenses, this mainly consists of secondary metabolites in plants that are resistant to *R. solanacearum*. These resistant metabolites disrupt the structure of *R. solanacearum* and inhibit their growth and reproduction. For example, coumarin inhibits acylhomoserine lactone synthesis, antagonizes quorum sensing (QS) regulatory proteins, and blocks receptor proteins in *R. solanacearum* (Qais et al. 2021). Daphnetin can inhibit extracellular polysaccharide (EPS) production and biofilm formation in *R. solanacearum* in vitro by repressing gene expression of *xanthomonas pathogenicity island regulatory protein R* (*xpsR*), *extracellular polysaccharide synthesis protein E* (*epsE*), *extracellular polysaccharide synthesis protein B* (*epsB*), and *lactobacillus extracellular matrix protein M* (*lexM*) (Yang et al. 2021b). 6-Methylcoumarin (Yang et al. 2021a, 2021b), 7-methoxycoumarin (Han et al. 2021), and hydroxycoumarins (Yang et al. 2016a, 2016b) were able to inhibit the expression of *filamenting temperature-sensitive mutant Z* (*ftsZ*), virulence-related genes *epsE*, *hypersensitive response and pathogenicity G* (*hrpG*) and *pseudomonas outer protein A* (*popA*), and *R. solanacearum* flagellar genes *flagellar sigma factor FliA* (*fliA*) and *flagellar transcriptional activator FlhC* (*flhC*), respectively, in *R. solanacearum*. In addition, coumarin analogs esculetin, umbelliferone, etc. can also disrupt the biofilm of *R. solanacearum* (Yang et al. 2016a, 2016b). In addition to coumarins, some other plant secondary metabolites, such as caffeic acid inhibits biofilm formation in *R. solanacearum* by suppressing the expression of *Lectin Mannose-Binding* (*lecM*) and *epsE* (Li et al. 2021). Methyl gallate can inhibit protein synthesis and succinate dehydrogenase. It can also inhibit the respiration of *R. solanacearum* and act as a fungicide (Fan et al. 2014).

Secondary metabolites of plants, such as flavonoids, also play an important role in combating biotic stresses. Flavonoids are a class of polyphenolic compounds widely found in plants and these compounds play an important role in plant growth and development and adaptation to adversity (Iwashina 2003; Pourcel et al. 2007). Studies have shown that flavonoids can not only improve the antioxidant capacity of plants and reduce the cell damage caused by oxygen radicals, but also induce the production of defense enzymes and related proteins, such as peroxidase and phenylalanine deaminase, to promote the defense response of plants (Zakaryan et al. 2017). In addition, flavonoids can act together with other bioactive compounds to enhance plant disease resistance (Wang et al. 2013). Flavonoids have been demonstrated to play an important role in resistance to bacterial wilt. For example, rutin and quercetin inhibit the growth of *R. solanacearum* by binding to the citrate synthase, disulfide coenzyme dehydrogenase, and F0F1 ATP synthase subunits of *R. solanacearum*, respectively (Hossain et al. 2021). Myricetin derivatives have also demonstrated promising antimicrobial activity against *R. solanacearum* (Zhong et al. 2017). The secondary metabolites produced by these plants play an important role in limiting the colonization and growth of pathogenic bacteria.

In our group, we previously produced a bacterial wilt-resistant mutant tobacco 486K by EMS mutagenesis of tobacco variety Cuibi-1 (CB-1). A highly bacterial wilt-resistant NIL, KCB-1, was generated by successive selfing and backcrossing. This experiment was conducted to investigate the functional role of naringenin, a metabolite specifically induced by KCB-1, with the aim of providing a reference for the breeding of resistance to *R. solanacearum* and plant protection.

## Results

### Root restriction of *R. solanacearum* colonization by KCB-1

CB-1 was subjected to EMS mutagenesis, while three generations of backcrossing and self-crossing produced KCB-1 (Supplementary Fig. S1). Indoor and field resistance tests of KCB-1 and CB-1 showed that KCB-1 is a highly resistant tobacco material to *R. solanacearum*.

**Figure 1.**
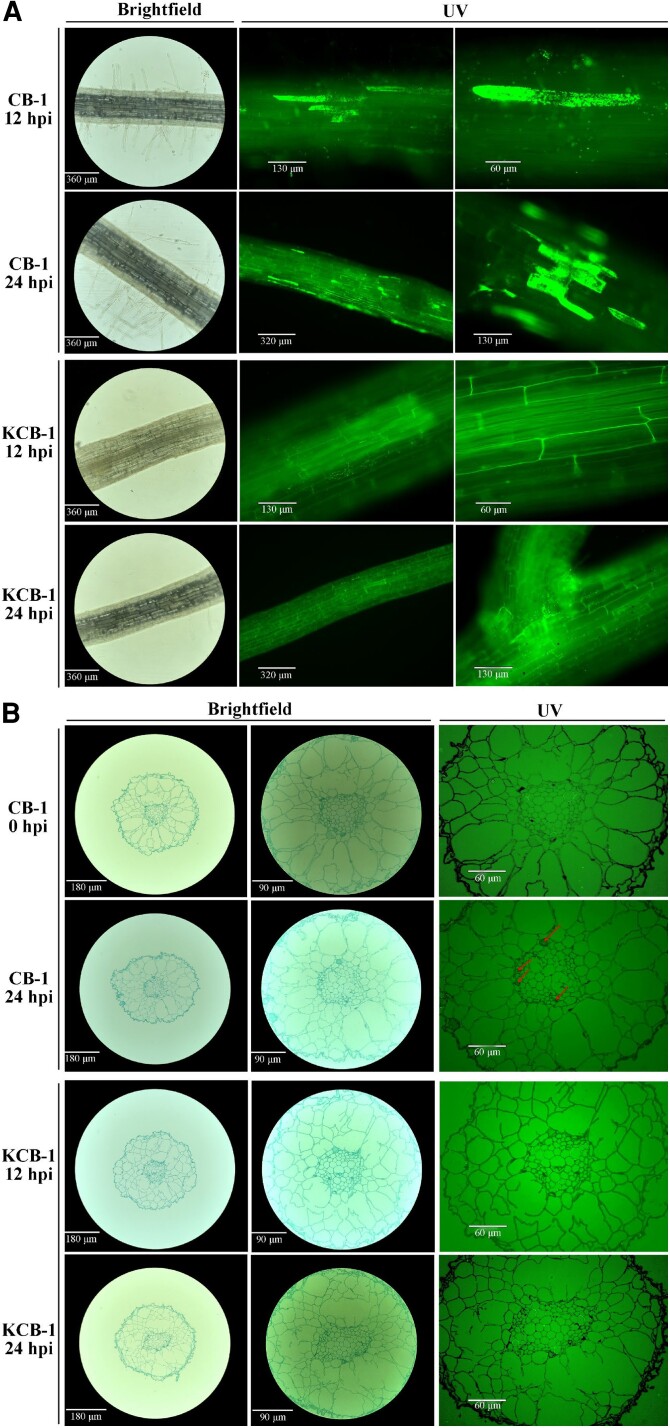
Colonization of roots of CB-1 and KCB-1 by *R. solanacearum*. **A)** Longitudinal colonization of roots of CB-1 and KCB-1 by *R. solanacearum* at 12 and 24 hpi. **B)** Cross-section of roots of CB-1 and KCB-1 at 0 and 24 hpi. hpi, hours post-infection.

At 12 and 24 h post-infection (hpi) of inoculation, we observed that *R. solanacearum* was heavily colonized in the roots of CB-1, while only a small amount of colonization was observed in KCB-1 (Fig. 1A). At 24 hpi, *R. solanacearum* colonization was further enhanced in CB-1, whereas it was still overall less than that of CB-1 in spite of an increase in the amount of colonization as well in KCB-1 (Fig. 1A). Higher colonization of *R. solanacearum* was observed only in the root crosses of KCB-1. In addition, at 24 hpi, root xylem conduits appeared clogged in CB-1 but not in KCB-1 (Fig. 1B). At 24 hpi, the number of *R. solanacearum* in the roots of both tobaccos was further counted by plate counting method. The results showed that the number of *R. solanacearum* in the roots of KCB-1 was significantly less than that of CB-1 (One-way ANOVA, *P <* 0.05) (Supplementary Fig. S2). This result suggests that the roots of KCB-1 have the ability to limit the colonization of *R. solanacearum*.

### *R. solanacearum* infestation specifically induces an increase in root naringenin content in KCB-1

When *R. solanacearum* infest tobacco roots, root chemical defense is another factor that may limit the colonization and growth of *R. solanacearum*. In the preliminary stage, we jointly analyzed the metabolomes and transcriptomes of KCB-1 and CB-1 roots before and after infestation with *R. solanacearum*. The results showed that the differential genes and metabolites of KCB-1 were mainly enriched in the phenylpropane biosynthesis pathway compared with CB-1 (Shi et al. 2022a, 2022b). Further, we analyzed the expression of genes related to the phenylpropane and flavonoid biosynthesis pathways. Genes related to the phenylpropane pathway, and the suberin biosynthesis pathway were differentially upregulated or downregulated in CB-1 and KCB-1 (Supplementary Fig. S3).

**Figure 2.**
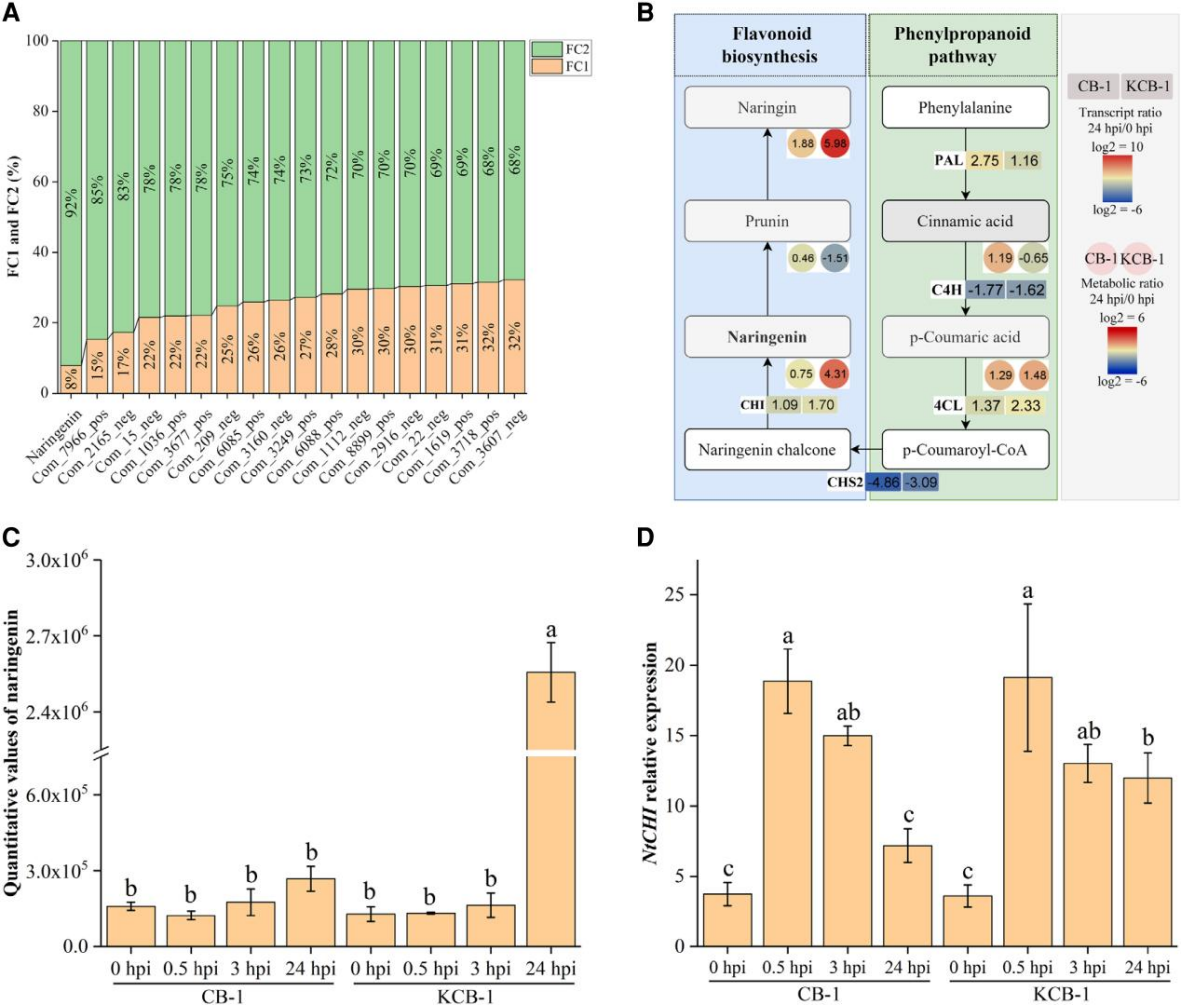
*Ralstonia solanacearum* infestation induces specific upregulation of naringenin content in KCB-1. **A)** Same differential metabolite content (FC2/FC1 ≥ 1.5) in CB-1_MD vs. CB-1_MA and KCB-1_MD vs. KCB-1_MA. FC1 represents the differential metabolite content ratio of CB-1_MD vs. CB-1_MA. FC2 represents the metabolite content ratio of KCB-1_MD vs. KCB-1_MA. **B)** Gene expression in the naringenin biosynthesis pathway and metabolite content. **C)** Naringin content in roots of KCB-1 and CB-1 at 0, 0.5, 3, and 24 hpi. **D)** Relative expression of the naringenin biosynthesis gene *NtCHI* by KCB-1 and CB-1 at 0, 0.5, 3, and 24 hpi. Letters between represent significant differences. hpi, hours post-infection, FC, fold change. Error bars represent standard deviation (Sd). Between letters represent significant differences (One-way ANOVA, *P* < 0.05).

Metabolomic analysis of KCB-1 and CB-1 before and after infestation with *R. solanacearum* showed that among the same differential metabolites in CB-1_MD vs. CB-1_MA and KCB-1_MD vs. KCB-1_MA, naringenin, a metabolite of the flavonoid biosynthesis pathway, was significantly induced to be produced in the roots of KCB-1 (One-way ANOVA, *P <* 0.05), whereas no significant change was observed in CB-1 (One-way ANOVA, *P* > 0.05) (Fig. 2, A to C). In addition, the expression of the naringenin biosynthesis-related gene, *NtCHI*, was significantly increased in KCB-1 at 0 to 24 hpi, whereas the expression of the *NtCHI* in CB-1 was not significantly changed at 24 hpi from 0 hpi (One-way ANOVA, *P* > 0.05), although it was also significantly increased at 0 to 3 hpi (Fig. 2D).

### Naringenin has an antagonistic effect on *R. solanacearum*

**Figure 3.**
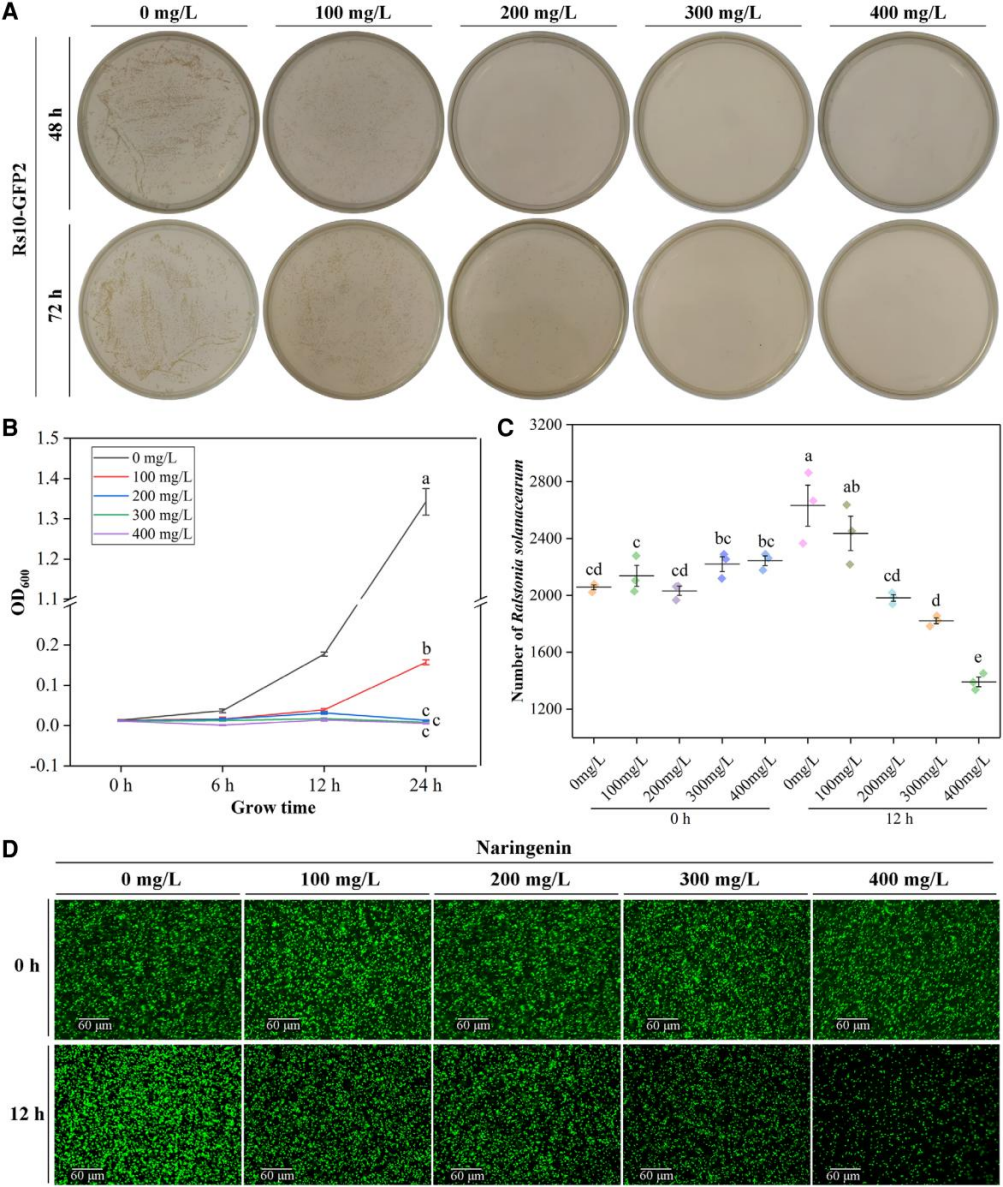
Inhibitory activity of naringenin against *R. solanacearum*. **A)** Growth of Rs10-GFP2 in NB medium with different concentrations of naringenin. Images were digitally extracted for comparison. **B)** Growth rate of *R. solanacearum* at 0 to 24 h in different concentrations of naringenin. **C)** Number of Rs10-GFP2 at 0 and 12 h under different concentrations of naringenin treatment. **D)** Imaging of Rs10-GFP2 under fluorescence microscope at 0 and 24 h with different concentrations of naringenin treatment. Notes: hours post-infection (hpi). Fold change (FC). Error bars represent standard deviation (Sd). Between letters represent significant differences (One-way ANOVA, *P* < 0.05). OD, optical density.

In order to verify whether naringenin has an inhibitory effect on *R. solanacearum*, we conducted an in vitro inhibition assay. The results showed that the minimum inhibitory concentration (MIC) and minimum bactericidal concentration (MBC) of naringenin against Rs-GFP2 were 200 and 300 mg/L, respectively (Fig. 3A). In addition, naringenin showed different inhibitory effects on other *R. solanacearum* variants Rs1, Rs5, Rs7, Rs9, and Rs_FJ from China (Supplementary Fig. S4, Supplementary Table S1). Evolutionary tree analysis of *R. solanacearum* variants from different countries showed that Y45, Rs1, Rs5, and Rs7 clustered together, and Rs_FJ and Rs9 clustered with JT523 and UW151, respectively (Supplementary Fig. S5, Supplementary Table S2). In addition, the growth rate assay showed that the OD_600_ value of *R. solanacearum* did not change at naringenin concentrations of 300 and 400 mg/L (Fig. 3B). Observation by fluorescence microscopy showed that the number of *R. solanacearum* was significantly reduced by the addition of naringenin at concentrations of 300 and 400 mg/L at 12 h (Fig. 3, C and D). In addition, we tested the effect of naringenin on the swimming motility of *R. solanacearum*, and the results showed that naringenin did not affect the swimming motility of *R. solanacearum* (Supplementary Fig. S6).

In order to further verify the control effect of naringenin on *R. solanacearum*, we watered naringenin solution at a concentration of 300 mg/L in CB-1 to observe the control effect of naringenin. The DI of CB-1 watered with naringenin was significantly decreased compared to CB-1 watered without naringenin (Supplementary Fig. S7). Therefore, we can conclude that naringenin is an important chemical defense component of resistant tobacco KCB-1 against *R. solanacearum*.

### Naringenin inhibits the growth and reproduction of *R. solanacearum* by destroying their structure

**Figure 4.**
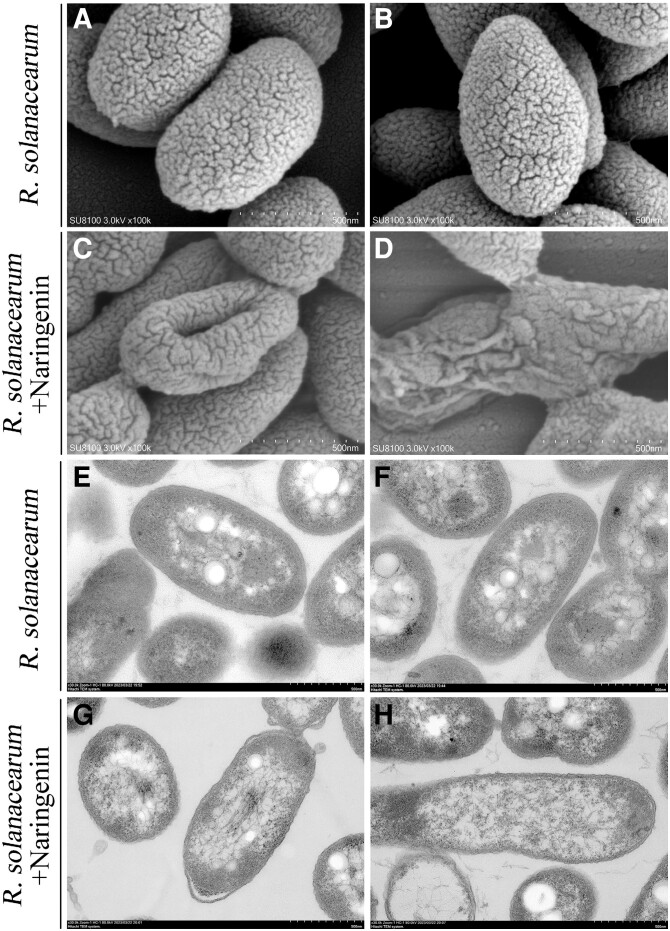
SEM and TEM images of naringenin-treated and untreated *R. solanacearum*. **A, B)** SEM images of *R. solanacearum* not treated with naringenin. **C, D)** SEM images of naringenin-treated *R. solanacearum*. **E, F)** TEM images of *R. solanacearum* not treated with naringenin. **G, H)** TEM images of naringenin-treated *R. solanacearum*.

In order to investigate the mechanism of naringenin inhibiting the growth and reproduction of *R. solanacearum*, we observed the morphology of *R. solanacearum* after the addition of naringenin by scanning electron microscopy (SEM) and transmission electron microscopy (TEM) (Fig. 4). SEM observation showed that the surface structure of the *R. solanacearum* without the addition of naringenin was intact (Fig. 4, A and B). The addition of naringenin resulted in depression of the cell wall of *R. solanacearum* and disruption of the cellular structure (Fig. 4, C and D). TEM observations showed that the cell wall and internal structure of untreated *R. solanacearum* were intact (Fig. 4, E and F). Whereas, naringenin treatment damages the cell wall structure of *R. solanacearum* (Fig. 4, G and H). This damage can lead to the death of *R. solanacearum*.

### *NtCHI* overexpression and knockdown restrict and do not restrict tobacco colonization to *R. solanacearum*, respectively

**Figure 5.**
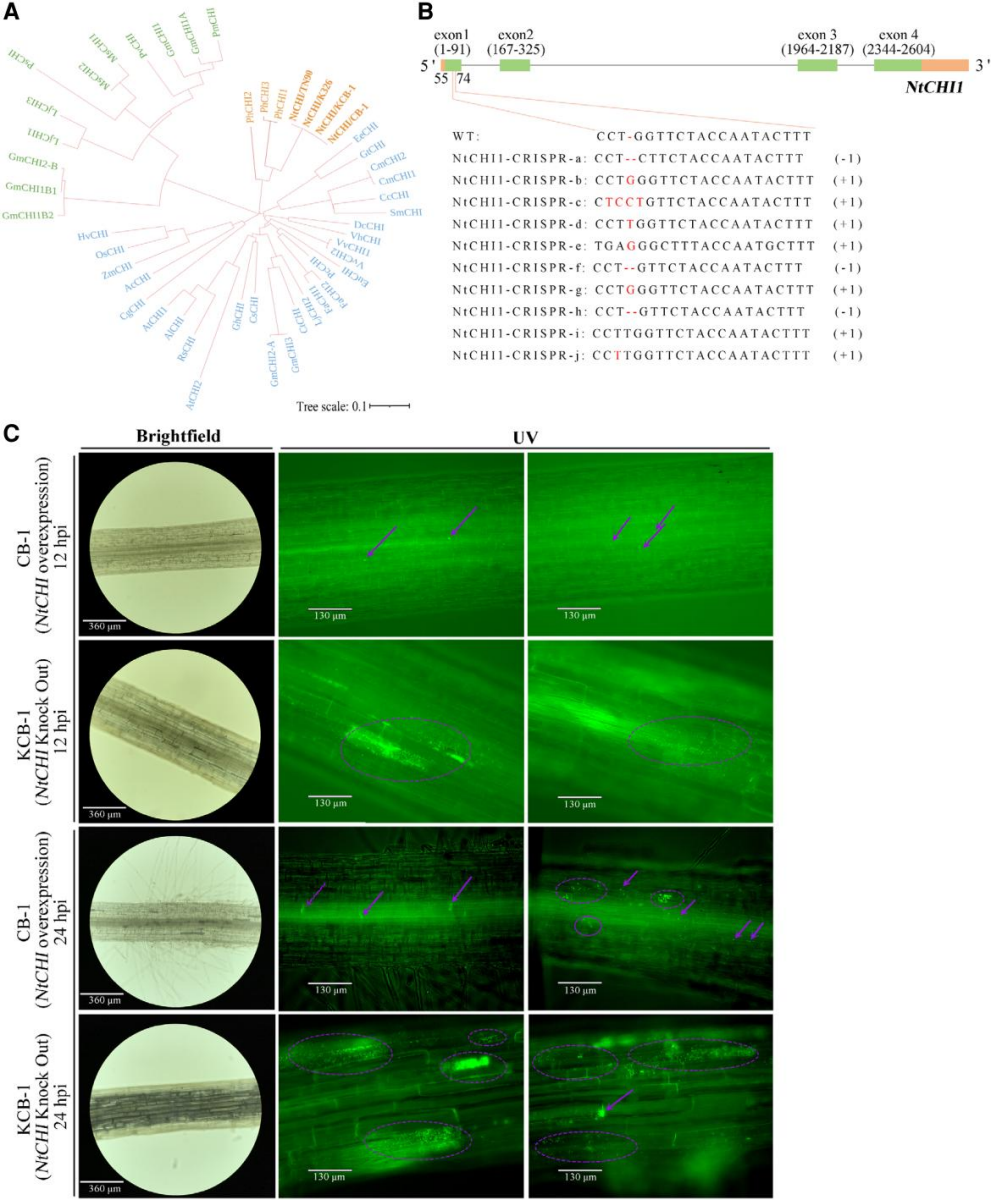
*NtCHI* knockdown and overexpression. **A)** Phylogenetic tree of *CHI* genes in each plant. **B)***NtCHI*-CRISPR gene structure. **C)***NtCHI* knockdown and overexpression materials. **C)***R. solanacearum* in *NtCHI* knockdown and overexpression materials at 12 and 24 hpi. WT, wild type; hpi, hours post-infection.

To verify the relationship between naringenin content and tobacco disease resistance, we knocked out and overexpressed *NtCHI*, a key gene for the synthesis of naringenin, in KCB-1 and CB-1, respectively. Sequencing results show that the sequences of *NtCHI* were the same in tobacco varieties TN90, K326, CB-1, and KCB-1 (Fig. 5A). The RT-qPCR results showed that the expression of *NtCHI* in the overexpressed material was significantly higher than that of KCB-1 and CB-1 (One-way ANOVA, *P* < 0.01, *P* < 0.01), respectively, when they were not inoculated with *R. solanacearum* (Supplementary Fig. S8). By sequencing the knockout material of KCB-1, the results showed a deletion or addition of one base in the first exon of *NtCHI*, which would result in a shifted mutation in the coding sequence of the gene later (Fig. 5B). At 12 hpi, *R. solanacearum* were present in only a small amount of colonization in the roots of the *NtCHI* overexpression material, whereas a large amount of colonization was present in the root cells of the *NtCHI* knockdown material (Fig. 5C). Further, at 24 hpi, a small amount of colonization by *R. solanacearum* was already present in the root xylem and surrounding cells of the *NtCHI* overexpressing material, while colonization was further intensified in the root cells of the *NtCHI* knockdown material (Fig. 5C).

### Naringenin induces a resistance response in tobacco to defend against *R. solanacearum*

**Figure 6.**
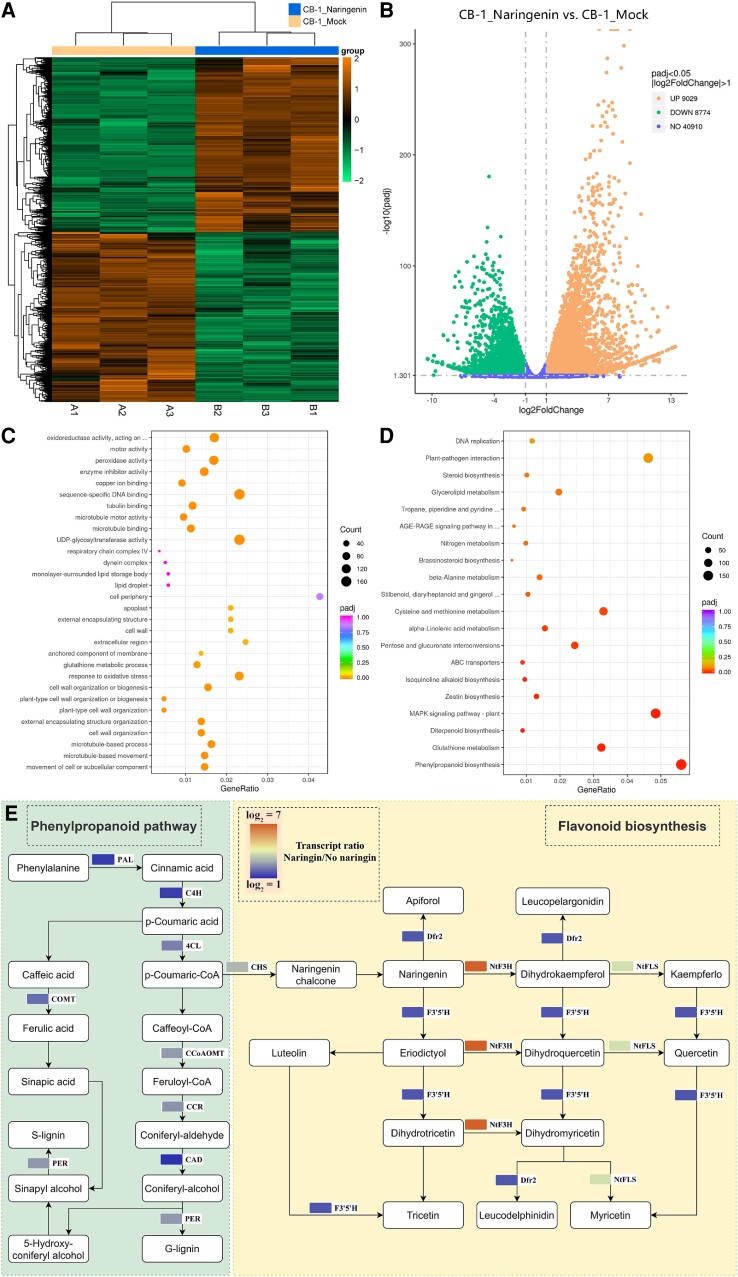
Naringenin activates the phenylpropane and flavonoid biosynthetic pathways of CB-1. **A)** Differential gene clustering of CB-1_Mock and CB-1_Naringenin. **B)** Volcano plot of up- and downregulated genes in CB-1_Mock vs. CB-1_Naringenin. **C)** GO enrichment analysis of CB-1_Mock vs. CB-1_Naringenin differential genes. **D)** KEGG enrichment analysis of CB-1_Mock vs. CB-1_Naringenin differential genes. **E)** Expression of genes related to phenylpropane and flavonoid biosynthesis pathways in CB-1_Mock and CB-1_Naringenin.

Naringenin inhibits the growth and reproduction of *R. solanacearum*. Whether naringenin then activates the plant's own resistance response was another question we wanted to explore. We then performed transcriptome sequencing on the roots of CB-1 without and with naringenin applied for 1 d. The results showed that the differentially expressed genes in the roots of CB-1 with and without naringenin applied had different expression patterns, respectively (Fig. 6A). There were 9,029 genes upregulated and 8,774 genes downregulated in CB-1 roots after naringenin application (Fig. 6B). This suggests that naringenin can lead to transcriptional reprogramming in CB-1 roots. GO enrichment analysis of the structure showed that the biological processes of the differential genes were closely related to the cell wall (Fig. 6C). The gene products perform their functions mainly in the cell wall, extracellular region. In addition, the molecular functions were also associated with pectinase activity, cellulose synthase activity, and xyloglucanosyltransferase activity. KEGG enrichment analysis showed that the phenylpropane as well as flavonoid biosynthesis pathways were activated by the application of naringenin (Fig. 6D). The expression of *Phenylalanine Ammonia Lyase* (*PAL*), *Cinnamic Acid 4-Hydroxylase* (*C4H*), *4-Coumarate-CoA Ligase* (*4CL*), *Catechol-O-Methyltransferase* (*COMT*), *Caffeoyl-CoA O-Methyltransferase* (*CCoAOMT*), *Cinnamoyl-CoA Reductase* (*CCR*), *Cinnamyl Alcohol Dehydrogenase* (*CAD*), and *Perillyl Alcohol Reductase* (*PER*) of the phenylpropane biosynthesis pathway was significantly upregulated (Fig. 6E). In addition, the expression of *Chalcone Synthase* (*CHS*), *Dihydroflavonol-4-Reductase 2* (*Dfr2*), *Flavonoid 3′,5′-Hydroxylase* (*F3′5'H*), and *Nicotiana tabacum Flavonol Synthase* (*NtFLS*) of the flavonoid biosynthesis pathway was significantly upregulated (Fig. 6E).

### Naringenin induces salicylic acid and reactive oxygen species accumulation in tobacco

How naringenin activates the pathway of CB-1 resistance to *R. solanacearum* is another question that needs to be investigated. Previous studies have shown that flavonoid-mediated pathogen resistance can be activated by salicylic acid (SA)-dependent pathways (Jia et al. 2010; Yang et al. 2016a, 2016b). In addition, there is a close association between SA accumulation and *R. solanacearum* resistance. For example, the *R. solanacearum* effector RipAY inhibits RipE1-mediated activation of the SA signaling pathway by degrading plant cell glutathione (Mukaihara et al. 2016; Sang et al. 2016, 2020). When naringenin was added, the SA biosynthesis-related gene *PAL1* was significantly upregulated and expressed in CB-1 roots (Student's *t*-test, *P* = 0.002), whereas no significant changes were observed for *EDS1*, *ICS1* (Student's *t*-test, *P* > 0.05, *P* > 0.05) (Supplementary Fig. S9A). This result suggests that naringenin regulates SA biosynthesis in tobacco roots through the mangiferic acid pathway. In addition, naringenin treatment decreased and increased the transcript levels of three and two pathogenesis-related *PR* genes, respectively (Supplementary Fig. S9B).

The genes associated with increased SA biosynthesis by naringenin prompted us to investigate whether naringenin increases the level of SA content in tobacco roots. Therefore, we measured the free SA content in CB-1 and KCB-1 after naringenin treatment. The results showed that naringenin significantly increased the free SA content in CB-1 and KCB-1 roots, respectively (One-way ANOVA, *P* < 0.05 and *P* < 0.05) (Supplementary Fig. S9C). In addition, SA content in roots of KCB-1 was significantly greater than that of CB-1 before and after naringenin addition at the same time (One-way ANOVA, *P* < 0.05 and *P* < 0.05) (Supplementary Fig. S9C). In addition, The SA content of roots of CB-1, KCB-1, and CB-1(OE) was significantly decreased after *R. solanacearum* (One-way ANOVA, *P* < 0.05, *P* < 0.05, and *P* < 0.05) (Supplementary Fig. S9D). However, after 24 hpi, the levels of root SA content in CB-1 (OE) were higher and lower than those in CB-1 and KCB-1, respectively (one-way ANOVA, *P* < 0.05 and *P* < 0.05) (Supplementary Fig. S9D).

In addition, *CAT1*, which regulates catalase activity, was significantly downregulated in CB-1 with the addition of naringenin compared to untreated CB-1 (Student's *t*-test, *P* = 0.009) (Supplementary Fig. S9A). We then examined the H_2_O_2_ and O^2−^ content of KCB-1 and CB-1 roots before and after naringenin treatment. When *Arabidopsis thaliana* was treated with naringenin, naringenin induced the accumulation of H_2_O_2_ and O^2−^, leading to a burst of reactive oxygen species (ROS) (An et al. 2021). We first treated CB-1 and KCB-1 with naringenin. The results showed that naringenin treatment led to a significant increase in H_2_O_2_ content in KCB-1 and CB-1 (One-way ANOVA, *P* < 0.05 and *P* < 0.05) (Supplementary Fig. S10A). And the level of H_2_O_2_ content in KCB-1 remained significantly higher than that in CB-1 after treatment (One-way ANOVA, *P* < 0.05 and *P* < 0.05) (Supplementary Fig. S10B). In addition, only the O_2_^−^ content level of CB-1 increased significantly after naringenin treatment (One-way ANOVA, *P* < 0.05) (Supplementary Figs. S10B and S11). In addition, the H_2_O_2_ content levels of CB-1, KCB-1, and CB-1(OE) were unchanged, increased, and rose, respectively, when *R. solanacearum* were treated (One-way ANOVA, *P* < 0.05, *P* < 0.05, and *P* < 0.05) (Supplementary Fig. S12A). In addition, the O_2_^−^ content levels of CB-1, KCB-1, and CB-1(OE) were significantly increased by *R. solanacearum* (One-way ANOVA, *P* < 0.05, *P* < 0.05, and *P* < 0.05) (Supplementary Fig. S12B). Pathogen infestation can induce a burst of ROS in plants. Whereas naringenin treatment of plants can likewise induce a burst of ROS, which seems to be advantageous for activating plant defense responses.

### *R. solanacearum* specificity induces upregulation of naringenin in the root secretion of resistant tobacco

In the previous part of the study, we identified the important role of the flavonoid naringenin for defense against *R. solanacearum*. Previous studies have also demonstrated that flavonoids are essential for the control of pathogenic bacteria. In view of this, we further investigated the characterization of root secretions of resistant and susceptible tobacco. We mainly performed targeted metabolomic assays for 35 flavonoids in the root secretions of KCB-1 and CB-1. Interestingly, however, at 6 dpi we detected naringenin only in the root secretions of KCB-1 and CB-1. Quantitative results showed that naringenin content was significantly downregulated and upregulated in the root secretions of CB-1 and KCB-1, respectively, before and after infestation with *R. solanacearum* (One-way ANOVA, *P* = 0.019, *P <* 0.001) (Supplementary Fig. S13). In addition, at 0 dpi, naringenin content in root secretions was significantly lower in KCB-1 than in CB-1 (One-way ANOVA, *P <* 0.001) (Supplementary Fig. S13). In contrast, at 6 hpi, naringenin content in root secretions of KCB-1 was significantly higher than that of CB-1 (One-way ANOVA, *P <* 0.001, *P <* 0.001) (Supplementary Fig. S13).

### Naringenin contributes extensively to the diversity of tobacco inter-root microorganisms

The secretion of plant metabolites into the body through the root system affects changes in inter-root microbes (Luo et al. 2020). Some specific root secretions convene beneficial microbial communities and inhibit the growth of pathogenic bacteria (Zhou et al. 2023). First, we verified the relationship between tobacco inter-root microbial communities and cycad resistance (Supplementary Fig. S14). We planted CB-1 in unsterilized and autoclaved field soils, and after inoculation with *R. solanacearum*, the incidence index of CB-1 grown in sterilized soil was significantly higher than that of the unsterilized (Student's *t*-test, *P* < 0.05) (Supplementary Fig. S14B). This result suggests that the inter-root bacterial community can influence tobacco resistance to *R. solanacearum*.

**Figure 7.**
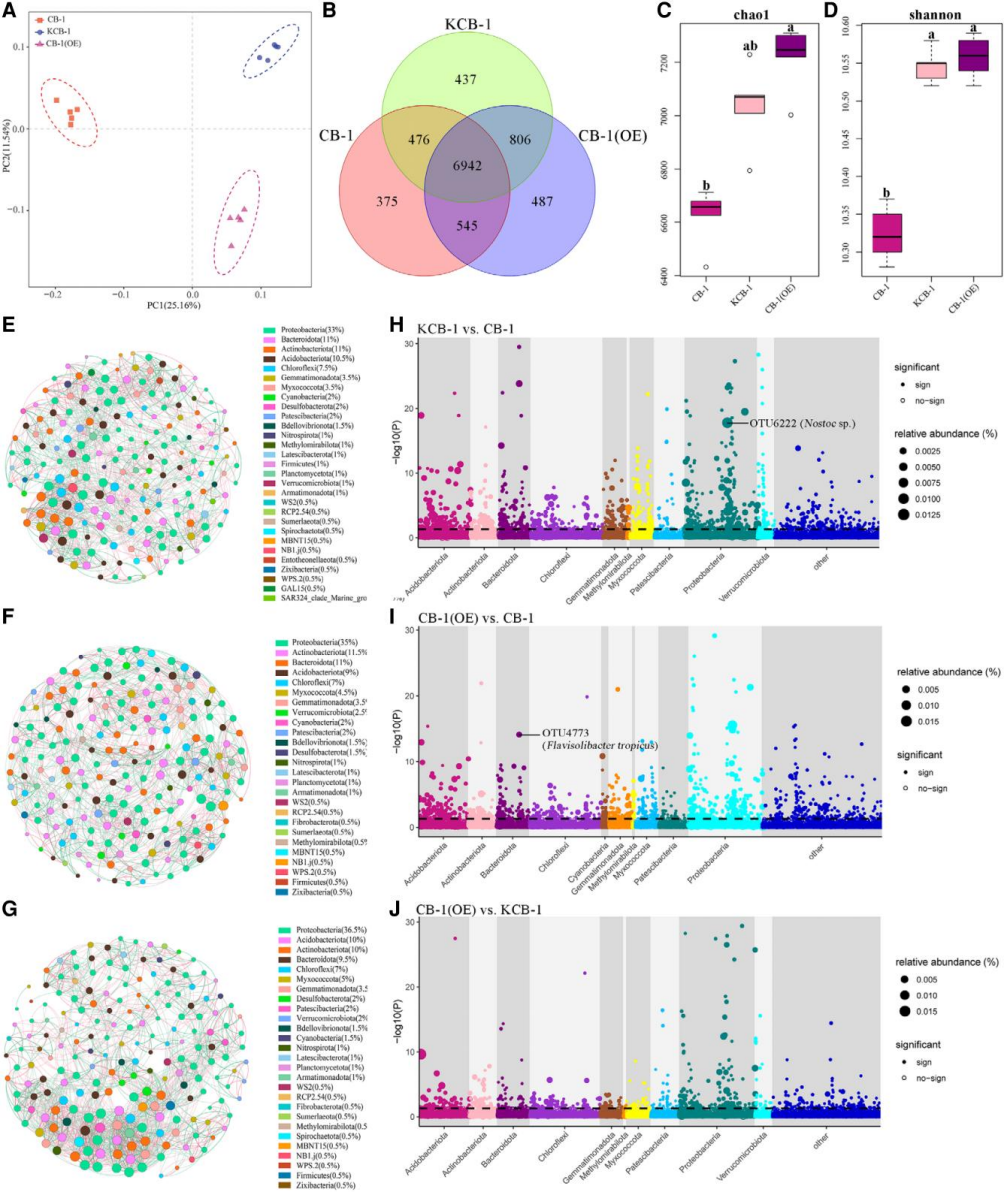
Inter-root bacterial communities of KCB-1, CB-1, and CB-1(OE). **A)** PCA analysis of KCB-1, CB-1, and CB-1(OE) inter-root bacterial communities. **B)** Venn diagram of the number of shared and exclusive OTUs of KCB-1, CB-1, and CB-1(OE) inter-root bacterial communities. **C)** Chao1 analysis plot of KCB-1, CB-1 and CB-1(OE) inter-root bacterial communities. **D)** Shannon analysis plot of KCB-1, CB-1, and CB-1(OE) inter-root bacterial communities. **E)** Network plot of the top 30 KCB-1 inter-root bacterial communities in abundance. **F)** Network plot of the top 30 CB-1 inter-root bacterial communities in abundance. **G)** Network plot of the top 30 CB-1 (OE) inter-root bacterial communities in abundance. **H)** Manhattan plot of OTUs enriched in KCB-1 inter-roots relative to CB-1. **I)** Manhattan plot of OTUs enriched in CB-1 (OE) inter-roots relative to CB-1. **J)** Manhattan plot of OTUs enriched in CB-1 (OE) inter-roots relative to KCB-1. The dashed line indicates the FDR-adjusted *P*-value significance threshold (*P* = 0.05). RA represents the relative abundance of OTU. PC, principal component; OE, overexpression.

Subsequently, we analyzed the inter-root bacterial communities of KCB-1, CB-1, and CB-1 (OE). The inter-group inter-root bacterial communities of KCB-1, CB-1, and CB-1 (OE) were highly differentiated (Fig. 7A). CB-1 (OE) had 7,748 and 7,487 shared OTUs with KCB-1 and CB-1, respectively, and 913 and 851 different OTUs (Fig. 7B). Chao1 and Simpson analysis showed that the diversity and richness of the inter-root bacterial community of CB-1 were significantly different from KCB-1 and CB-1 (OE), respectively (One-way ANOVA, *P* < 0.05, *P* < 0.05) (Fig. 7, C and D). In contrast, the diversity and abundance of inter-root bacterial communities of KCB-1 and CB-1(OE) were not significantly different (One-way ANOVA, *P* > 0.05, *P* > 0.05) (Fig. 7, C and D). By comparing the network diagrams of the inter-root bacterial communities of KCB-1, CB-1, and CB-1(OE), the results showed that the networks of the inter-root bacterial communities of both CB-1(OE) and KCB-1 were more complex than that of CB-1 (Fig. 7, E to G). Furthermore, among all known cultured bacteria, OTU6222 (*Nostoc* sp.) and OUT4773 (*Flavisolibacter tropicus*) were the most abundant species in KCB-1 and CB-1(OE), respectively, compared to CB-1 (Fig. 7, H and I). In contrast, no known cultured bacteria were seen in the top 50 abundance strains in CB-1(OE) compared to KCB-1 (Fig. 7J).

Further, we exogenously applied naringenin in the soil where CB-1 was grown. High-throughput sequencing of the inter-root bacterial communities with and without naringenin addition showed that the inter-root bacterial communities were equally different between the groups of CB-1 and CB-1 (+Naringenin) (Supplementary Fig. S15A). there were 10,755 and 3,574 differentiated OUTs of CB-1 and CB-1 (+Naringenin), respectively and 6,119 shared OUTs (Supplementary Fig. S15B). Chao1 and Simpson analyses showed that CB-1 and CB-1 (+Naringenin) were significantly different in diversity and richness, respectively (Student’s *t*-test, *P* = 0.002, *P* = 0.001) (Supplementary Fig. S15, C and D). In addition, by comparing the network maps of the top 20 inter-root bacterial communities in terms of abundance of CB-1 and CB-1 (+Naringenin), the results showed that the addition of naringenin resulted in a more complex and tightly knit network of inter-root bacterial communities in CB-1 (Supplementary Fig. S15, E and F). degree increased from 2,914 to 3,608. furthermore, the CB-1 (+Naringenin) rhizosphere bacterial community had 57.48% of positive interactions and 42.52% of negative interactions with each other. In contrast, CB-1 had only 49.55% of positive interactions and 50.45% of negative interactions among rhizobacterial communities. In addition, *Pseudomonas* sp. (OTU239) in particular was the most abundant and significantly enriched OTU in CB-1 (+Naringenin) (Tukey's HSD test, *P* = 0.008) (Supplementary Fig. S15G).

### Naringenin as a marker of resistance in bacterial wilt-resistant tobacco

Significantly elevated naringenin content in bacterial wilt-resistant tobacco after inoculation with *R. solanacearum* is one of the keys to the difference between resistant and susceptible tobacco (One-way ANOVA, *P <* 0.05). We speculated whether there is a qualitative relationship between different levels of resistant and susceptible tobacco and the amount of naringenin when it is induced. To test this hypothesis, we measured the changes in naringenin content in the roots of CB-1, KCB-1, HD, K326, and YY97 before and after inoculation with *R. solanacearum*. The results showed that after inoculation with *R. solanacearum*, the root naringenin content of YY97 and K326, which are moderately resistant to *R. solanacearum*, was significantly greater than that of CB-1 and HD, which are susceptible (One-way ANOVA, *P <* 0.05) (Supplementary Figs. S16 to S18). In contrast, the resistant mutant KCB-1 had significantly (One-way ANOVA, *P <* 0.05) greater naringenin content than the other four materials both before and after inoculation (Supplementary Fig. S15). In addition, the naringenin content of YY97, CB-1, and HD was not altered by *R. solanacearum* infestation. In contrast, KCB-1 and K326 showed significant changes in naringenin content in roots infested with *R. solanacearum* (One-way ANOVA, *P <* 0.05, *P <* 0.05) (Supplementary Fig. S16). This result indicated that there was a significant association between tobacco resistance to *R. solanacearum* and root naringenin content. Therefore, we concluded that naringenin can be used as a resistance marker for resistant tobacco.

## Discussion

In this study, naringenin content was specifically upregulated in the roots of resistant mutant tobacco after *R. solanacearum* infestation, whereas it was unchanged in the roots of susceptible tobacco CB-1. As verified by in vitro function, naringenin inhibited the growth and reproduction of *R. solanacearum*. In addition, naringenin activated the expression of genes related to the phenylpropane and flavonoid biosynthesis pathways in tobacco. The phenylpropane pathway is important for strengthening the cell wall. The flavonoid biosynthesis pathway, on the other hand, is important for limiting the growth and reproduction of *R. solanacearum* (Shi et al. 2023). In addition, the experimental results demonstrated that naringenin regulates SA biosynthesis in tobacco roots through the mangiferolic acid pathway. External application of naringenin resulted in a significant upregulation of tobacco SA content (Supplementary Fig. S9C). Moreover, in resistant tobacco, SA content was high at 24 hpi, which is undoubtedly crucial for activating the immune response of tobacco against *R. solanacearum*.

The prominent role played by naringenin in biotic stress is increasingly emphasized. Naringenin has been shown to inhibit the growth of tobacco blight mold (Sun et al. 2022), *Magnaporthe oryzae* (Chen et al. 2022), *Pseudomonas syringae* (An et al. 2021) and *Pseudomonas aeruginosa* (Vandeputte et al. 2011), among others. In *P. aeruginosa*, naringenin affects the growth of *P. aeruginosa* by decreasing the production of pyocyanin and elastase in *P. aeruginosa* (Vandeputte et al. 2011). Moreover, naringenin reduced the expression of several QS-controlled genes in *P. aeruginosa* (Vandeputte et al. 2011). When applied externally, naringenin activates plant resistance responses to pathogenic bacteria. In tobacco, naringenin enhances resistance to tobacco blight (*Phytophthora nicotianae*) mainly by inducing the accumulation of ROS, the expression of genes related to SA biosynthesis, and the underlying pathogen resistance genes *PR1* and *SAR8.2* (Sun et al. 2022). In *Arabidopsis*, naringenin first activates the production of ROS, which in turn activates the expression of resistance genes through two pathways (accumulation of SA and activation of MPK), respectively, contributing to *Arabidopsis* resistance to *P. syringae* (An et al. 2021). Naringenin also promotes colonization of the intercellular space of wheat roots by *Azospirillum brasilense* and other diazotrophic bacteria (Webster et al. 1998). In addition, naringenin has been shown to stimulate Herbaspirillum in *Arabidopsis* to colonize the lateral root cell interstitium (Webster et al. 1998). In view of the important role of naringenin for biotic stresses, it is important to investigate in depth the role of naringenin in resistance to pathogenic bacteria for the control of plant diseases.

Flavonoids play an increasingly important role in plant disease resistance. They act both as antimicrobial agents and as inducers of plant defense responses (Päsold et al. 2010; Zhuang et al. 2013; Deryabin et al. 2019). For example, barley produces saponins that inhibit the spread of *Bromus diandricus* (Bouhaouel et al. 2019). Hambelin and baicalein in the root secretion of *Scutellaria baicalensis* have inhibitory effects against rice rice blast fungus (*M. oryzae*) and *R. solanacearum*, respectively (Do et al. 2021). In addition, treatment of *Arabidopsis* with quercetin (Jia et al. 2010) or naringenin (An et al. 2021) induced a reactive oxygen burst in *Arabidopsis* and enhanced resistance to *P. aeruginosa*.

In this study, we characterized and quantified root secretions of resistant and susceptible tobacco before and after inoculation. <***>At 6 h, we detected the presence of naringenin only among 35 flavonoid substances. This might be the reason for the short collection time of root secretions. However, this does not negate the important role of changes in naringenin content in root secretions in the fight against *R. solanacearum*. The naringenin content of root secretion of resistant tobacco KCB-1 was significantly lower than that of susceptible tobacco CB-1 when it was not inoculated with *R. solanacearum* (One-way ANOVA, *P <* 0.05). After inoculation with *R. solanacearum*. the naringenin content of root secretion of resistant tobacco was significantly upregulated (One-way ANOVA, *P <* 0.05). However, naringenin content in root secretions of susceptible tobacco was significantly downregulated (One-way ANOVA, *P <* 0.05). This might be due to the inhibition of naringenin biosynthesis in the roots of susceptible tobacco by *R. solanacearum*. The results of the pot experiment showed that exogenous application of naringenin significantly reduced the incidence index of tobacco bacterial wilt (One-way ANOVA, *P <* 0.05). This suggests that naringenin has some application for the control of *R. solanacearum*.

Flavonoids also affect the abundance and complexity of plant rhizosphere microbes. In this study, we demonstrated that the diversity and abundance of the inter-root bacterial community of susceptible tobacco CB-1 were significantly smaller than those of KCB-1 and CB-1 (OE), respectively (One-way ANOVA, *P* < 0.05, *P* < 0.05). In contrast, there were no significant differences in the diversity and abundance of the inter-root bacterial communities of KCB-1 and CB-1 (OE) (One-way ANOVA, *P* > 0.05, *P* > 0.05). In addition, the network complexity of the inter-root bacterial community was higher in CB-1 (OE) than in CB-1. Therefore, the differences in the abundance, diversity, and network complexity of the inter-root bacterial community might have influenced the differences between the resistances to some extent. In addition, *Nostoc* sp. and *F. tropicus* as the most abundant bacterial colonies in KCB-1 vs. CB-1 and CB-1 (OE) and CB-1, respectively, might be important for the control of bacterial wilt. In addition, we demonstrated that exogenous application of naringenin led to significant changes in the diversity and abundance of bacterial communities in the CB-1 root system (Student's *t*-test, *P* < 0.05). In particular, *Pseudomonas* sp. was significantly enriched in CB-1 (+Naringenin) (Student's *t*-test, *P* < 0.05). It has been shown that *Pseudomonas* sp. strain CHA0 has a strong inhibitory effect on *R. solanacearum* (Clough et al. 2022). The 2,4-diacetylphloroglucinol, pyoluteorin, and orfamides A and B secondary metabolite gene clusters were present in CHA0. And all of these compounds are inhibitory to *R. solanacearum*. When CHA0 was applied externally, it significantly reduced the incidence of blight in the tomato variety “Micro-Tom” (Student's *t*-test, *P* < 0.05) (Clough et al. 2022). In addition, other *Pseudomonas* strains, such as CLP-6, produce volatile organic compounds (VOCs) that are inhibitory to *R. solanacearum* under acidic conditions. The VOCs produced by this strain can assist in the control of tobacco bacterial wilt or other blight diseases of lycopersicon crops in acidic soils (Zhao et al. 2023). In addition, it was found that strains of the genus *Pseudomonas* were more effective than the genus Bacillus in controlling bacterial wilt (Chandrasekaran et al. 2016). Moreover, some of the strains of this genus also have growth-promoting effects on plants (Chandrasekaran et al. 2016). Therefore, some strains of the genus *Pseudomonas* might be important for the control of tobacco bacterial wilt, but this needs further validation.

**Figure 8.**
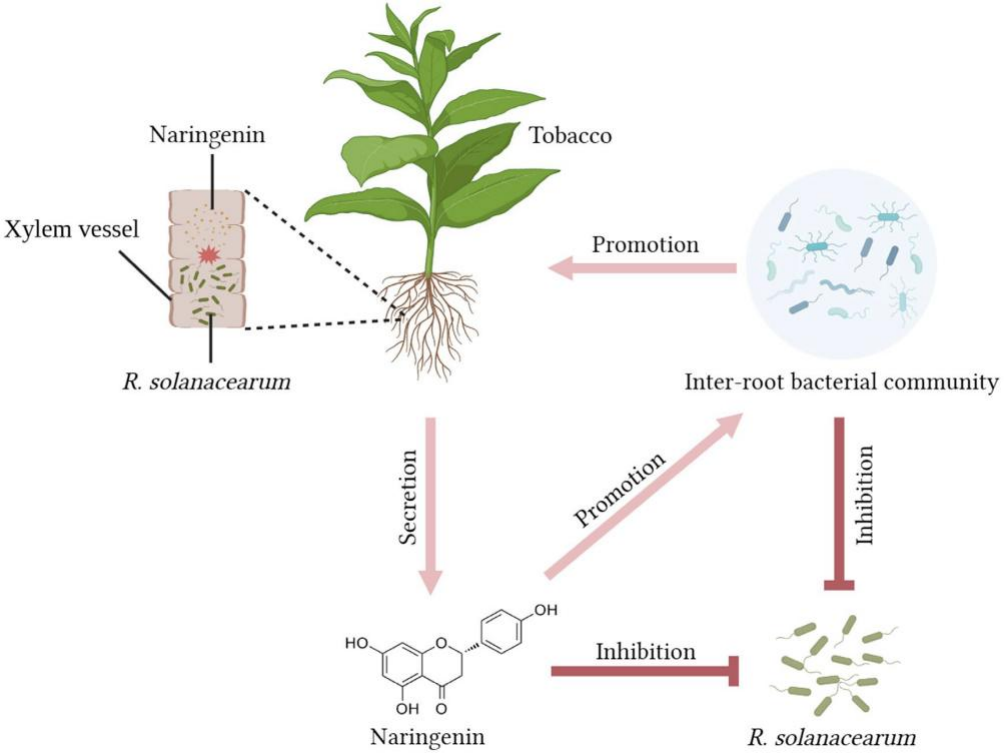
Network diagram of resistance mechanisms in cycad-resistant tobacco. After *R. solanacearum* blight infestation, resistant tobacco KCB-1 produces naringenin through the flavonoid biosynthesis pathway, which restricts the growth and colonization of *R. solanacearum* blight in the roots of KCB-1. Resistant tobacco KCB-1 affects the abundance of *R. solanacearum* in the environment by secreting naringenin into the environment. Naringenin secreted by KCB-1 into the environment can affect the inter-root bacterial community in KCB-1, leading to an increase in the abundance of some strains of bacteria that are antagonistic to *R. solanacearum*. And some strains can promote tobacco growth.

In this study, we demonstrated the functional role of naringenin, a metabolite that is specifically upregulated in resistant tobacco. This led us to consider how foreign aid application of naringenin reduces the incidence index of tobacco bacterial wilt. First, externally applied naringenin activates tobacco's own resistance response, which is necessary for tobacco to resist infestation by *R. solanacearum*. Second, naringenin itself can inhibit the growth and reproduction of *R. solanacearum*. And naringenin applied externally can inhibit the population of *R. solanacearum* in the soil. Third, we consider that naringenin can affect the tobacco inter-root bacterial community. Whereas, foreign aid application of naringenin resulted in a more aggregated and complex network of tobacco inter-root bacterial communities and an increase in the abundance of antagonistic bacteria (Fig. 8). These antagonistic bacteria are essential for limiting the colonization of *R. solanacearum*.

## Materials and methods

### Plant materials and resistance evaluation

The plant materials included K326 (moderately resistant to bacterial wilt), Hongda (HD) (susceptible to bacterial wilt), YY97 (moderately resistant to bacterial wilt), Cuibi-1 (CB-1) (susceptible to bacterial wilt), and KCB-1 (a NIL of CB-1, highly resistant to bacterial wilt). Seeds of each tobacco material were planted in greenhouse trays and grown at 30 °C under a 16 h light/8 h dark cycle. Tobacco plants were inoculated with *R. solanacearum* (OD_600_ = 1.0) after 2 mo of growth. The *R. solanacearum* used for inoculation was the green fluorescent protein-labeled strain Rs10-GFP2 (Kanamycin B resistant). Strain Rs10-GFP2 was purified from diseased tobacco in Shandong. Wilt symptoms were scored using a scale from 0 to 4. 0 = healthy plants without wilt, 1 = 25%, 2 = 50%, 3 = 75% of leaves wilted, and 4 = complete wilt. The disease index (DI) was calculated by averaging the disease scores for each tobacco (*n* > 10) (Sebastià et al. 2021).

### Detection of *R. solanacearum* content in roots

Fresh root samples of 0.1 g of CB-1 and KCB-1 were taken at 24 hpi and ground in a mortar and pestle with three replicates of each material. After grinding, they were transferred into 10 mL centrifuge tubes and serially diluted 1 × 10^8^ times with ddH_2_O. Fifty microliter was taken and spread evenly on NA medium. Addition of Kanamycin B (the final concentration is 50 *μ*g/mL) to NA medium. After 24 h of incubation in a constant temperature incubator at 28 °C, the colonies of *R. solanacearum* were counted using ImageJ software (Chen et al. 2014).

### Paraffin section and staining observation

The roots of KCB-1 and CB-1 were fixed with tissue fixative for 24 h. Refer to Supplementary Method S1 for detailed methodology.

### Microscopy observation

Lateral roots of CB-1 and KCB-1 infested with *R. solanacearum* were taken at 12 and 24 hpi and placed on slides, 20 *μ*L of ddH_2_O was added dropwise, and the coverslips were covered. And then the distribution of *R. solanacearum* was observed under a forward inverted integrated fluorescence microscope (Echo Laboratories, USA). For paraffin sectioned samples, they were directly observed under a forward inverted integrated fluorescence microscope.

### SEM and TEM

Naringenin (concentration of 300 mg/L) treated and untreated *R. solanacearum* were collected by centrifugation. Refer to Supplementary Method S2 for detailed methodology.

### RT-qPCR

Total RNA was extracted from tobacco material in total III with TRIzol reagent. cDNA was reverse transcribed using a reverse transcription kit quantitative real-time polymerase chain reaction (RT-qPCR). Ntubc2 was selected as the internal reference gene (Supplementary Table S3) (Schmidt and Delaney 2010).

### Compounds

Naringenin (HPLC ≥ 98%, w/v) (Acmec, Shanghai) was dissolved in 0.5 mL of anhydrous ethanol and prepared to final concentrations of 100, 200, 300, and 400 mg/L. The dissolved naringenin was added to NA medium to prepare naringenin suspensions at different concentrations. The same volume of anhydrous ethanol was added to the control.

### MIC and MBC determination

MIC and MBC were determined using the agar coefficient method (Han et al. 2021). A range of final concentrations included 100, 200, 300, and 400 mg/L. Fifty microliter of diluted *R. solanacearum* suspension (OD_600_ = 0.05) was spread on agar plates containing Kanamycin B (the final concentration is 50 *μ*g/mL). The inoculated media were incubated in an incubator at 28 °C for 48 and 72 h. Three replicates were set up for all assays.

### Growth curve of *R. solanacearum* after naringenin treatment

See Supplementary Method S3 for details.

### Swimming ability of *R. solanacearum* under naringenin treatment

See Supplementary Method S4 for details.

### Evaluation of naringenin for the control of tobacco bacterial wilt

Naringenin solutions were subjected to watering tests to assess the effectiveness of naringenin in the control of bacterial wilt (Han et al. 2021). Refer to Supplementary Method S5 for detailed operational steps and assessment methods.

### Phylogenetic analysis of *R. solanacearum* variants

See Supplementary Method S6 for details.

### Phylogenetic analysis of *chalcone isomerase* (*CHI*) genes

See Supplementary Method S7 for details.

### *NtCHI* knockdown and overexpression vector construction

*NtCHI* was overexpressed and knocked down using pCAMBia35S-EGFP and pMV-U6sg-BsaI vectors, respectively. For detailed steps, refer to Supplementary Method S8.

### Transcriptome sequencing

When CB-1 grew to 2 mo old, three CB-1 were treated with no and applied naringenin (at a concentration of 300 mg/L), respectively, for 1 d. And then the roots were collected for transcriptome sequencing. Sequencing samples included the control CB-1_Mock as well as the treatment group CB-1_Naringenin. Three replicates each of control and treatment groups, CB-1_Mock-A1, CB-1_Mock-A2, CB-1_Mock-A3 and CB-1_Naringenin_B1, CB-1_Naringenin_B2, CB-1_Naringenin_B3. Total RNA were extracted from the roots of six tobacco plants using the kit, and the transcriptomes were sequenced by Novogene. The raw data after sequencing were analyzed with reference to Shi et al. (2022a, 2022b).

### Determination of naringenin content

Three replicates each of root samples of CB-1, KCB-1, HD, K326, and YY97 at 0 and 1 d of infestation with *R. solanacearum* were collected. All materials used were 2-mo-old tobacco seedlings grown in a greenhouse. Individual samples were taken at 0.1 g fresh weight. Naringenin was extracted from tobacco roots using a naringenin extraction kit (Solarbio, Beijing). For detailed extraction and characterization of naringenin, please refer to Supplementary Method S9.

### Collection and identification of root secretions

Root secretions were collected from KCB-1 and CB-1 at 0 and 6 dpi, respectively, and 35 flavonoid metabolites were detected (Supplementary Table S4). For detailed sampling steps, refer to Supplementary Method S10.

### SA content assay

CB-1, KCB-1, and CB-1(*NtCHI* overexpression (OE)) were each grown in a greenhouse until 2 mo of age. Individual samples were taken at 0.1 g fresh weight. The roots of three CB-1 and KCB-1 plants were taken after 1 d treatment without and with naringenin (300 mg/L), respectively. And then the SA content of tobacco roots was detected by SA content assay kit (Lai Er Bio-Tech, Hefei). In addition, three plants each of CB-1, KCB-1, and CB-1 (OE) were taken before and after *R. solanacearum* treatment, and then the SA content of tobacco roots was determined as described above. For detailed sampling steps, refer to Supplementary Method S11.

### Determination of H_2_O_2_ and O^2−^ content

CB-1, KCB-1, and CB-1(OE) were hydroponic seedlings grown 2-mo-old in the greenhouse. Roots of each of the three materials were treated with *R. solanacearum* for 1 d and the control with NB medium. Individual samples were taken at 0.1 g fresh weight. The contents of H_2_O_2_ and O^2−^ were detected with H_2_O_2_ and O^2−^ detection kits (Beijing Solarbio Science & Technology Co., Ltd, Beijing). The same methodology was used for tests to detect H_2_O_2_ and O^2−^ before and after treatment of CB-1 and KCB-1 with naringenin. For detailed extraction procedure refer to Supplementary Method S12.

### 16S sequencing

Genomic DNA was extracted from inter-root soil samples using the E.Z.N.A. Soil DNA Kit (Omega Bio-tek, Inc., USA). Refer to Supplementary Method S13 for detailed extraction and sequencing steps.

### Data analysis

Data were analyzed using SPSSAU (https://spssau.com/). Graphing was done using Origin software. Significance of differences between test groups was analyzed by one-way ANOVA for multiple comparisons or one-way comparisons using Tukey's HSD test or the unpaired Student's *t*-test, respectively. Statistical significance was found at *P <* 0.05.

### Accession numbers

Sequence data from this article can be found in the GenBank data libraries under the following accession numbers: *NtCHI*, NM_001325287; NtCHI, NP_001312216.

## Supplementary Material

kiae185_Supplementary_Data

## Data Availability

The data underlying this article are available in the article and in its online supplementary material.

## Supplementary data

The following materials are available in the online version of this article.

**Supplementary Figure S1.** Process of breeding 486K and CB-1 into improved line KCB-1 after three generations of backcrossing and self-crossing and identification of resistance in the field.

**Supplementary Figure S2.** Colonization of CB-1 and KCB-1 roots by *R. solanacearum*.

**Supplementary Figure S3.** Changes in phenylpropanes, flavonoids, and corky biosynthesis pathway genes and metabolites of KCB-1 and CB-1 at 0 and 24 hpi.

**Supplementary Figure S4.** Growth of Rs_FJ, Rs5, Rs7, Rs9, and Rs11 in NB petri dishes supplemented with naringenin at concentrations of 0, 100, 200, 300, and 400 mg/L at 48 and 72 h, respectively.

**Supplementary Figure S5.** Clustering map of egl gene sequences of *R. solanacearum*.

**Supplementary Figure S6.** Swimming motility of *R. solanacearum* on NB medium supplemented with different concentrations of naringenin.

**Supplementary Figure S7.** Onset of CB-1 with and without naringenin.

**Supplementary Figure S8.** Relative expression of *NtCHI* in KCB-1, CB-1, and CB-1 (Overexpression).

**Supplementary Figure S9.** Changes in the expression of genes related to SA biosynthesis and changes in content in tobacco roots.

**Supplementary Figure S10.** Changes in the content of H_2_O_2_ and O^2−^ in roots after naringenin treatment of KCB-1 and CB-1.

**Supplementary Figure S11.** Standard sample calibration curve for sodium nitrite solution.

**Supplementary Figure S12.** Changes in the content of H_2_O_2_ and O^2−^ in roots after *R. solanacearum* treatment of KCB-1, CB-1, and CB-1(OE).

**Supplementary Figure S13.** Naringenin content in root secretions of KCB-1 and CB-1 at 0 and 6 hpi of inoculation with *R. solanacearum*. Letters represent significant differences between them.

**Supplementary Figure S14.** Effect of sterilized and nonsterilized soil on CB-1 incidence and growth.

**Supplementary Figure S15.** Effect of naringenin on tobacco inter-root bacterial communities.

**Supplementary Figure S16.** Changes in naringenin content in roots of CB-1, KCB-1, HD, K326, and YY97 before and after inoculation with *R. solanacearum*.

**Supplementary Figure S17.** HPLC assay of naringenin in tobacco roots.

**Supplementary Figure S18.** Standard sample calibration curve for naringenin.

**Supplementary Table S1.** Minimum inhibitory concentration and minimum lethal concentration of naringin on *R. solanacearum*.

**Supplementary Table S2.** Sequence information of the *egl* gene of *R. solanacearum*.

**Supplementary Table S3.** Primer sequences covered in the article.

**Supplementary Tables S4.** Thirty-five flavonoids in targeted metabolomic assays.

**Supplementary Method S1.** Paraffin section and staining observation.

**Supplementary Method S2.** Scanning electron microscopy (SEM) and transmission electron microscopy (TEM).

**Supplementary Method S3.** Growth curve of *R. solanacearum* after naringenin treatment.

**Supplementary Method S4.** Swimming ability of *R. solanacearum* under naringenin treatment.

**Supplementary Method S5.** Evaluation of naringenin for the control of tobacco bacterial wilt.

**Supplementary Method S6.** Phylogenetic analysis of *R. solanacearum* variants.

**Supplementary Method S7**. Phylogenetic analysis of *chalcone isomerase* (*CHI*) genes.

**Supplementary Method S8.** *NtCHI* knockdown and overexpression vector construction.

**Supplementary Method S9.** Determination of naringenin content.

**Supplementary Method S10.** Collection and identification of root secretions.

**Supplementary Method S11.** SA content assay.

**Supplementary Method S12.** Determination of H_2_O_2_ and O^2−^ content.

**Supplementary Method S13.** 16S sequencing.

## Dive Curated Terms

The following phenotypic, genotypic, and functional terms are of significance to the work described in this paper:

CAT1 Gramene: AT1G20630
CAT1 Araport: AT1G20630

PAL1 Gramene: AT2G37040
PAL1 Araport: AT2G37040

naringenin CHEBI: CHEBI:50202

flavonoids CHEBI: CHEBI:72544

C4H Gramene: AT2G30490
C4H Araport: AT2G30490

EDS1 Gramene: AT3G48090
EDS1 Araport: AT3G48090

PR1 Gramene: AT2G14610
PR1 Araport: AT2G14610

CHS Gramene: AT5G13930
CHS Araport: AT5G13930
