# Supplementary material for: Naringenin restricts the colonization and growth of Ralstonia solanacearum in tobacco mutant KCB-1
Source: Plant Physiol. 2024 Apr 4;195(3):1818–34. doi: 10.1093/plphys/kiae185 (PMC11213252; doi:10.1093/plphys/kiae185)
Supplement: kiae185_Supplementary_Data [file kiae185_supplementary_data.pdf]

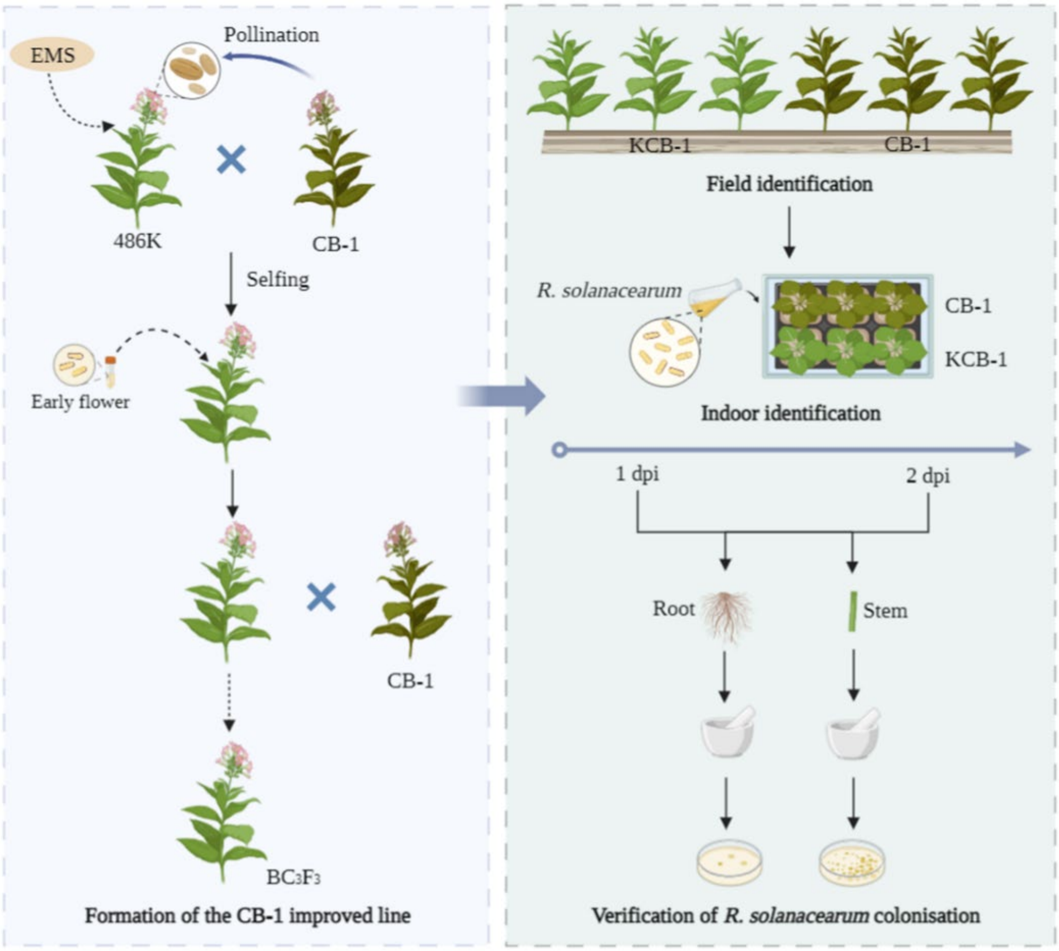

Supplemental Figure S1 Process of breeding 486K and CB-1 into improved line KCB-1 after three generations of backcrossing and self-crossing and identification of resistance in the field. Ethyl Methanesulfonate (EMS). Days post-infection (dpi).

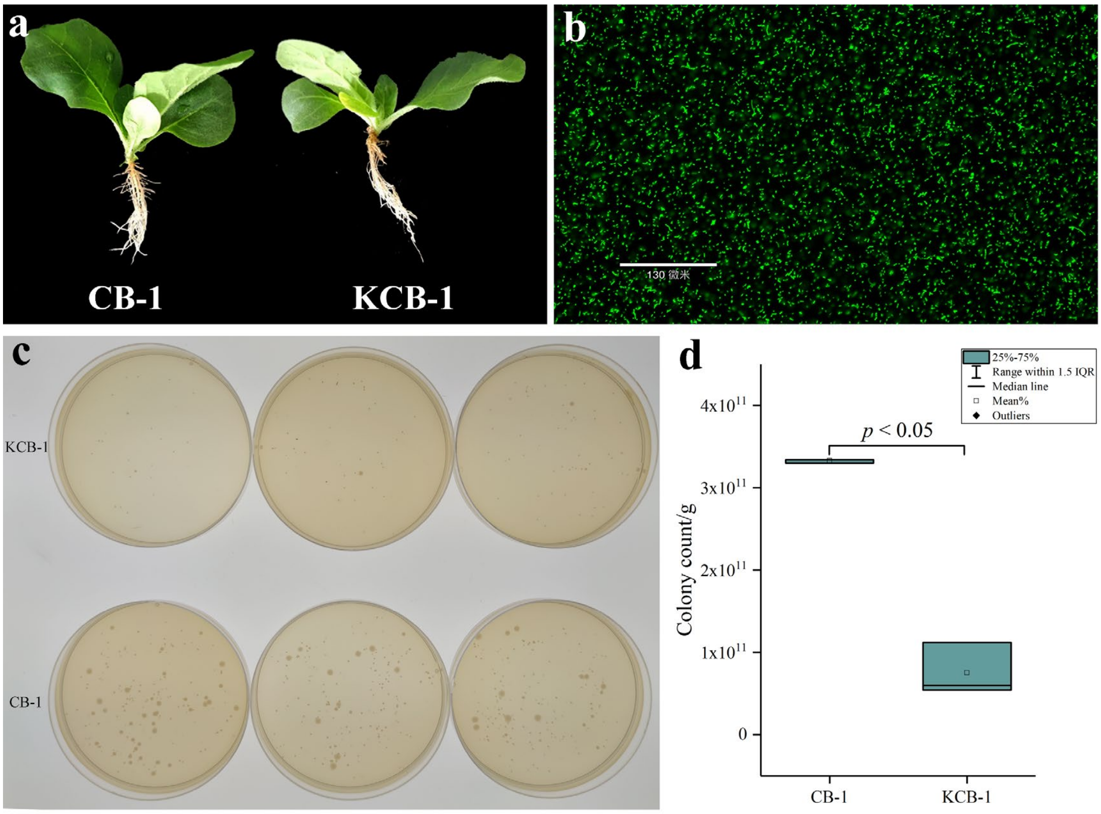

Supplemental Figure S2 Colonization of CB-1 and KCB-1 roots by *R. solanacearum*. a. CB-1 and KCB-1 seedlings. The growth of CB-1 and KCB-1 roots on NB medium at 24 hpi. b. Imaging of *R. solanacearum* strain Rs10-GFP2 under fluorescence microscope. c. Growth of *R. solanacearum* on NB medium at 24 hpi in CB-1 and KCB-1 roots. d. Counts of *R. solanacearum* colonizing CB-1 and KCB-1 roots at 24 hpi. e. The growth of *R. solanacearum* on NB medium in CB-1 and KCB-1 roots at 24 hpi in CB-1 and KCB-1 roots at 24 hpi in CB-1 and KCB-1 (Student's t test, $p < 0.05$). Note: a. is a digital extraction of images for comparison.

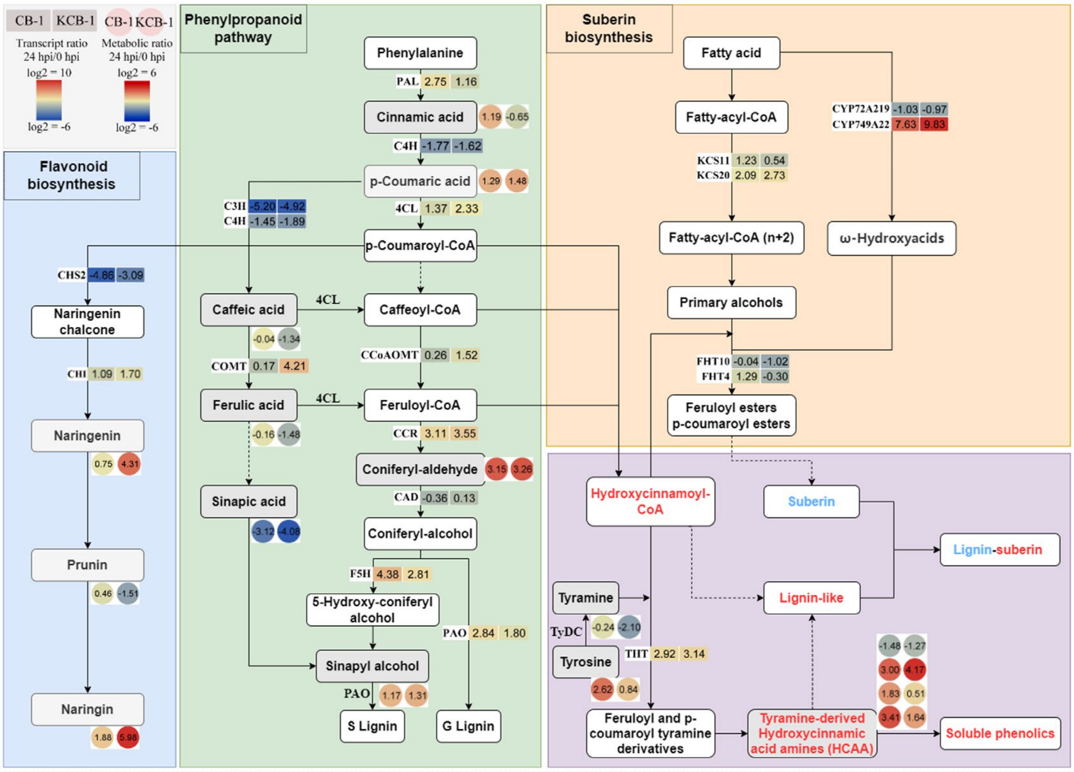

Supplemental Figure S3 Changes in phenylpropanes, flavonoids, and corky biosynthesis pathway genes and metabolites of KCB-1 and CB-1 at 0 and 24 hpi.

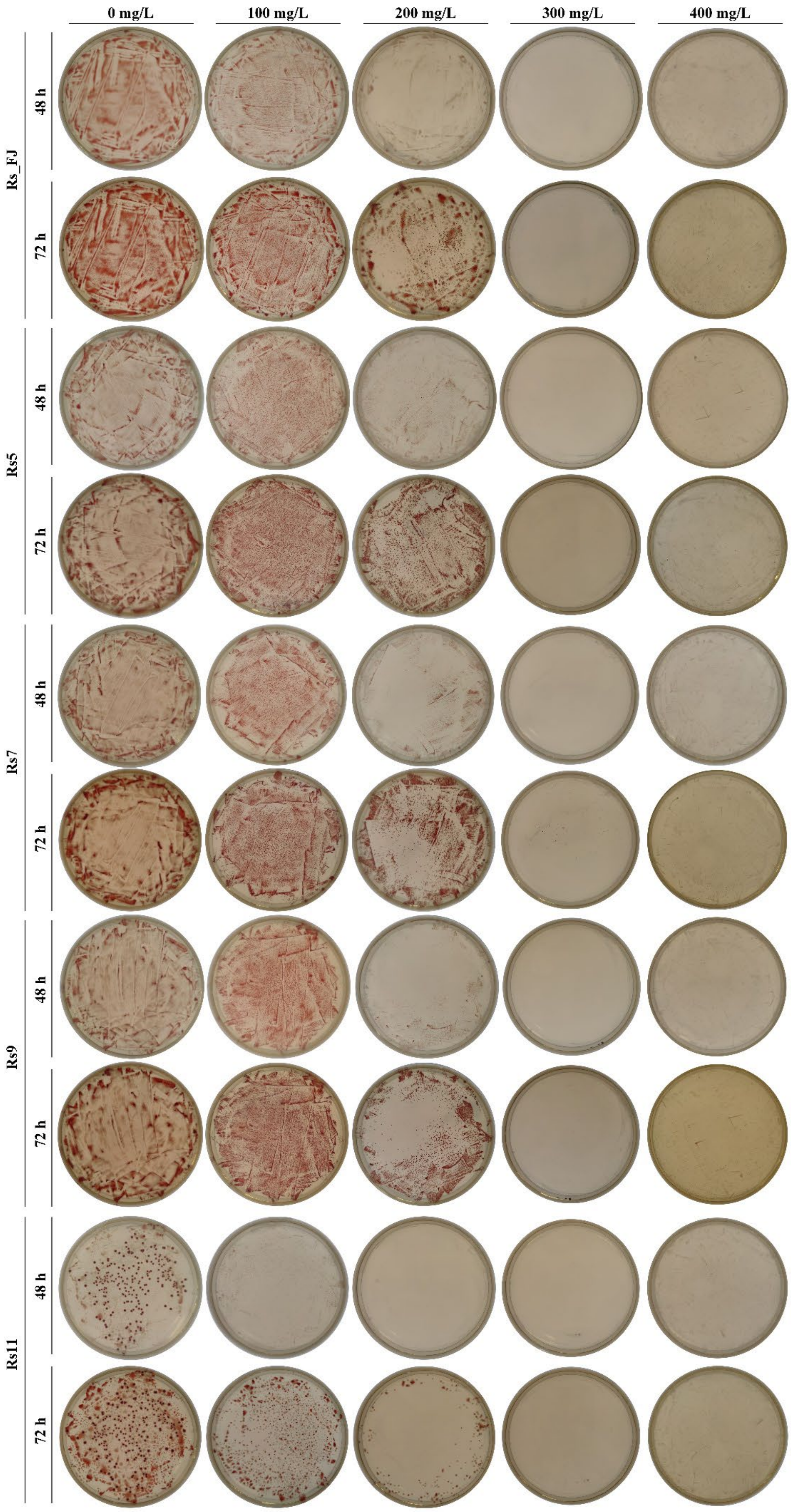

0 mg/L
100 mg/L
200 mg/L
300 mg/L
400 mg/L
Rs_FJ
Rs5
Rs7
Rs9
Rs11
48 h
72 h

Supplemental Figure S4 Growth of Rs_FJ, Rs5, Rs7, Rs9 and Rs11 in NB petri dishes supplemented with naringenin at concentrations of 0, 100, 200, 300 and 400 mg/L at 48 and 72h, respectively. Images were digitally extracted for comparison.

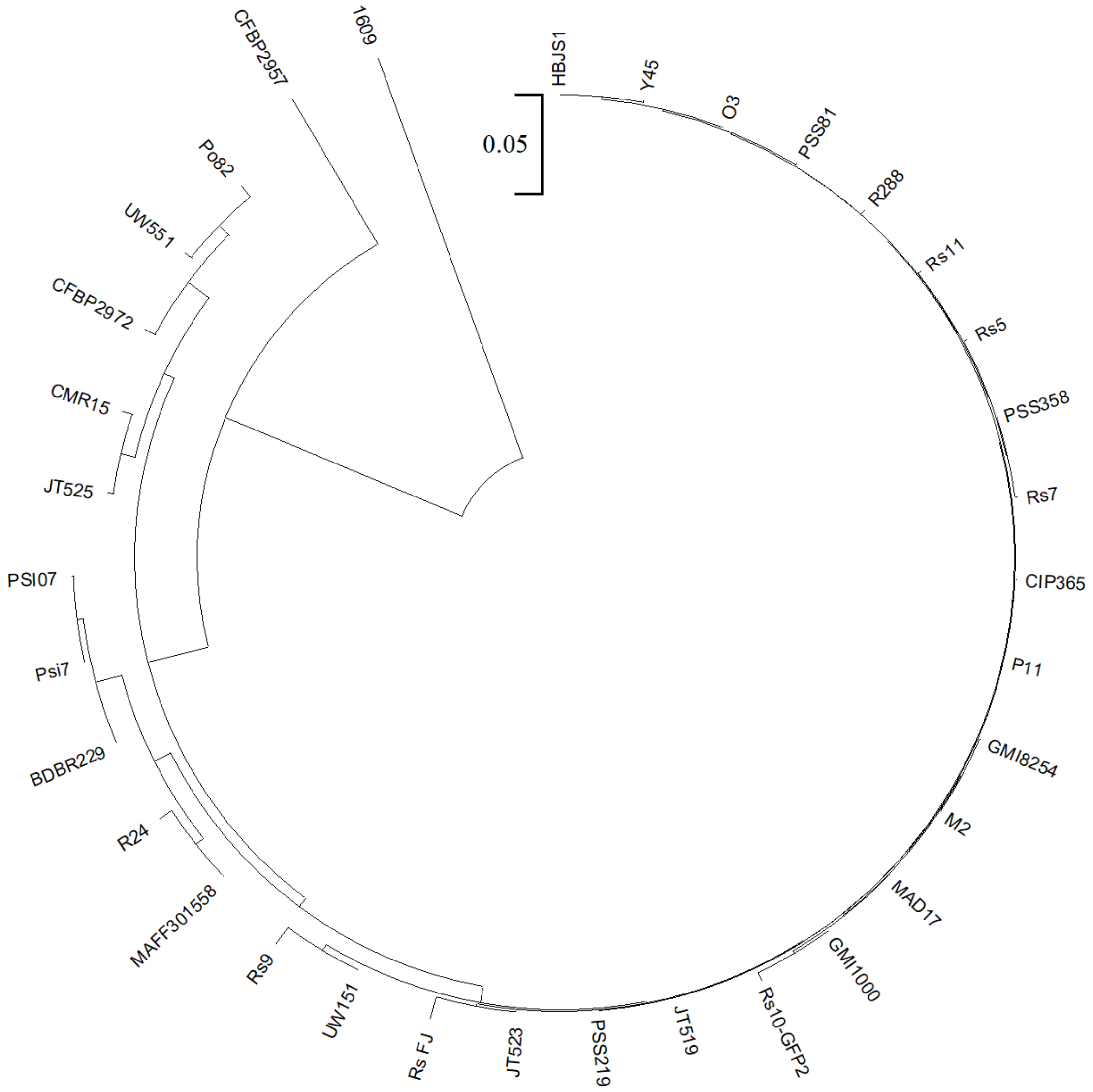

Supplemental Figure S5 Clustering map of egl gene sequences of *R. solanacearum*.

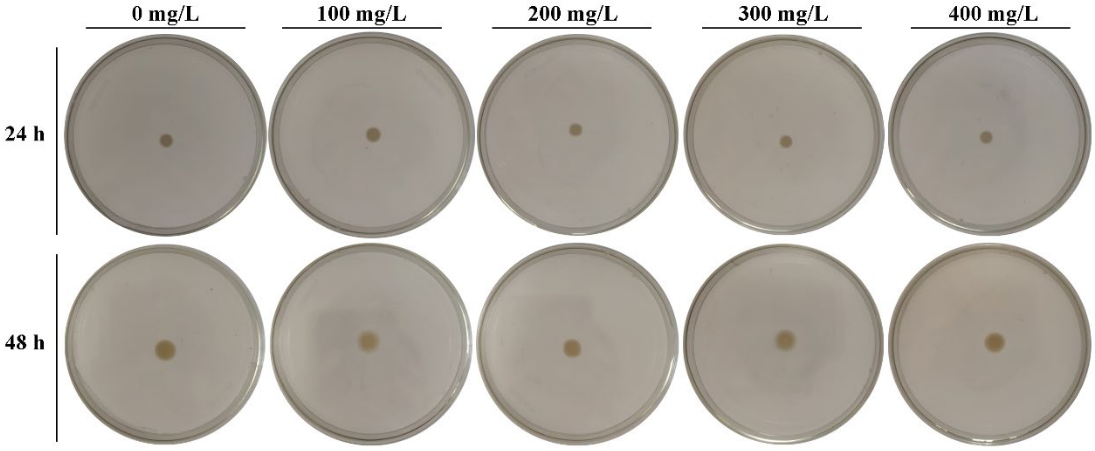

Supplemental Figure S6 Swimming motility of *R. solanacearum* on NB medium supplemented with different concentrations of naringenin. Images were digitally extracted for comparison.

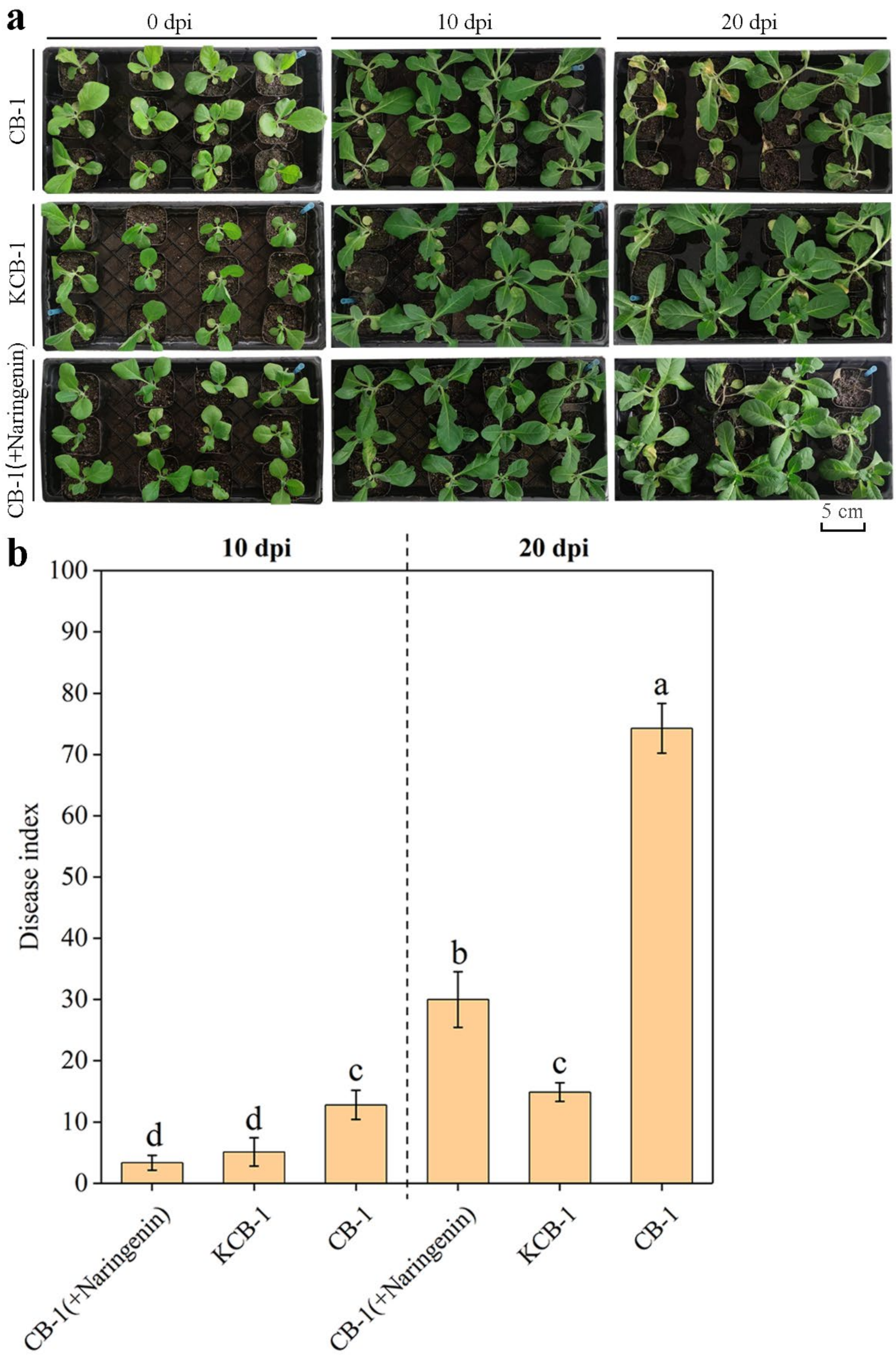

Supplemental Figure S7 Onset of CB-1 with and without naringenin. a. Incidence of CB-1, KCB-1 and CB-1 (+Naringenin) at 0, 10 and 20 dpi. b. Incidence indices of CB-1, KCB-1, and CB-1 (+Naringenin) at 10 and 20 dpi. Notes: days post-infection (dpi). Error bars represent Standard Deviation (SD). Between letters represent significant differences (One-way Anova, $p < 0.05$).

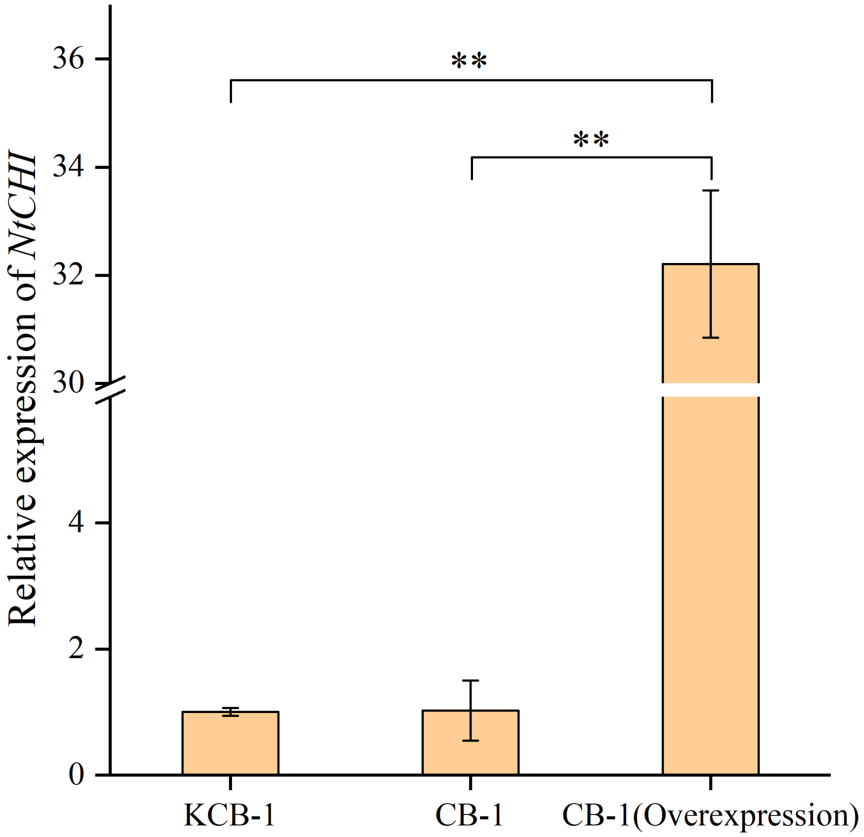

Supplemental Figure S8 Relative expression of *NtCHI* in KCB-1, CB-1 and CB-1 (Overexpression). Double asterisks represent $p < 0.01$ (One-way Anova). Error bars represent Standard Deviation (SD).

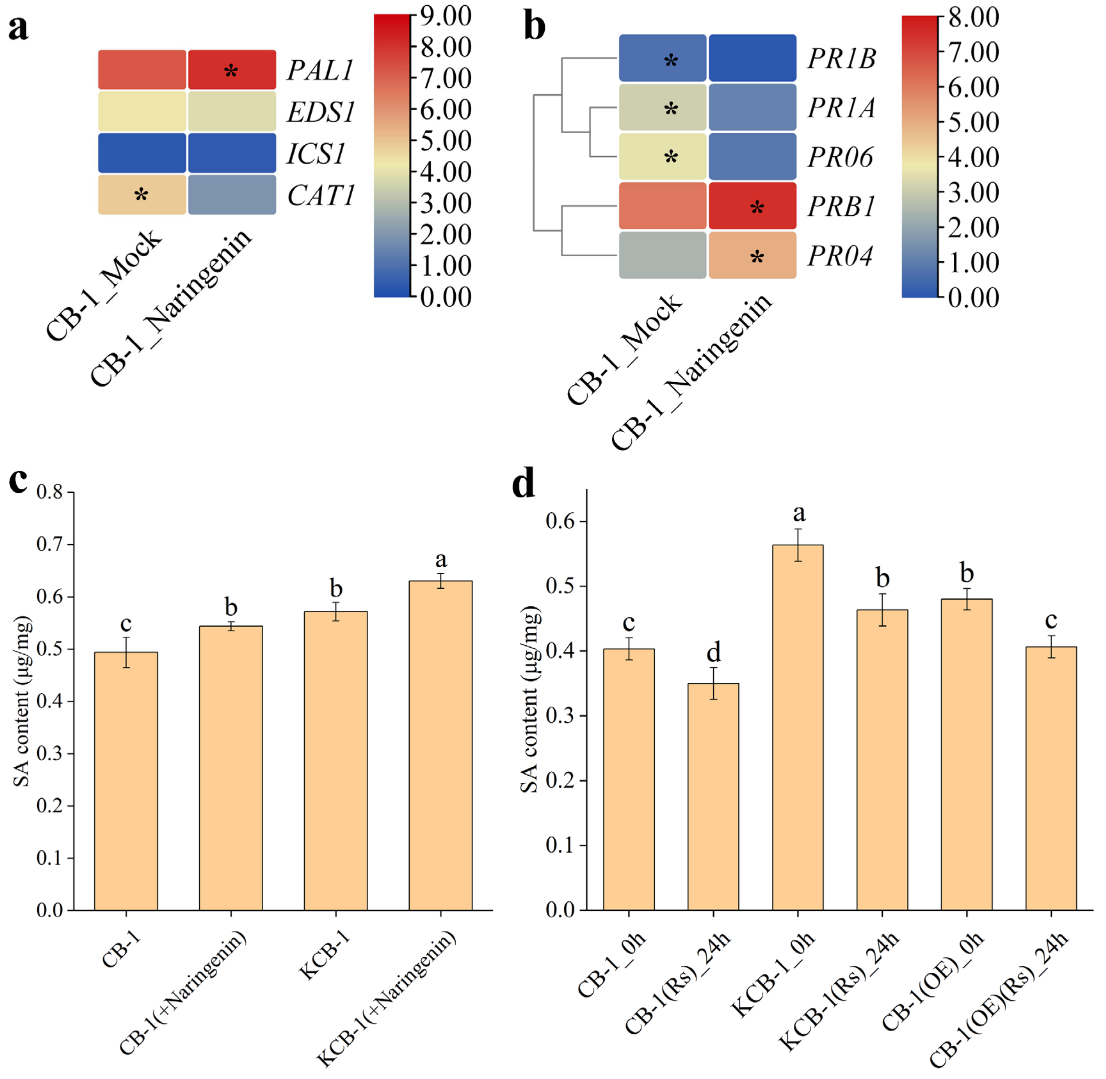

Supplemental Figure S9 Changes in the expression of genes related to salicylic acid (SA) biosynthesis and changes in content in tobacco (*Nicotiana tabacum*) roots. a. Gene expression of SA biosynthesis pathway in CB-1 before and after naringenin treatment. b. Expression of *PR1B*, *PR1A*, *PR06*, *PRB1* and *PR04* genes in CB-1 before and after naringenin treatment. c. Changes in the content of SA in KCB-1 and CB-1 at 0 and 24 h after naringenin treatment. d. Changes in the content of SA in KCB-1, CB-1 and CB-1 (OE) at 0 and 24 hpi after *R. solanacearum* treatment. Notes: asterisk represents $p < 0.05$ (Student's t test). Error bars represent Standard Deviation (SD). Between letters represent significant differences (One-way Anova, $p < 0.05$).

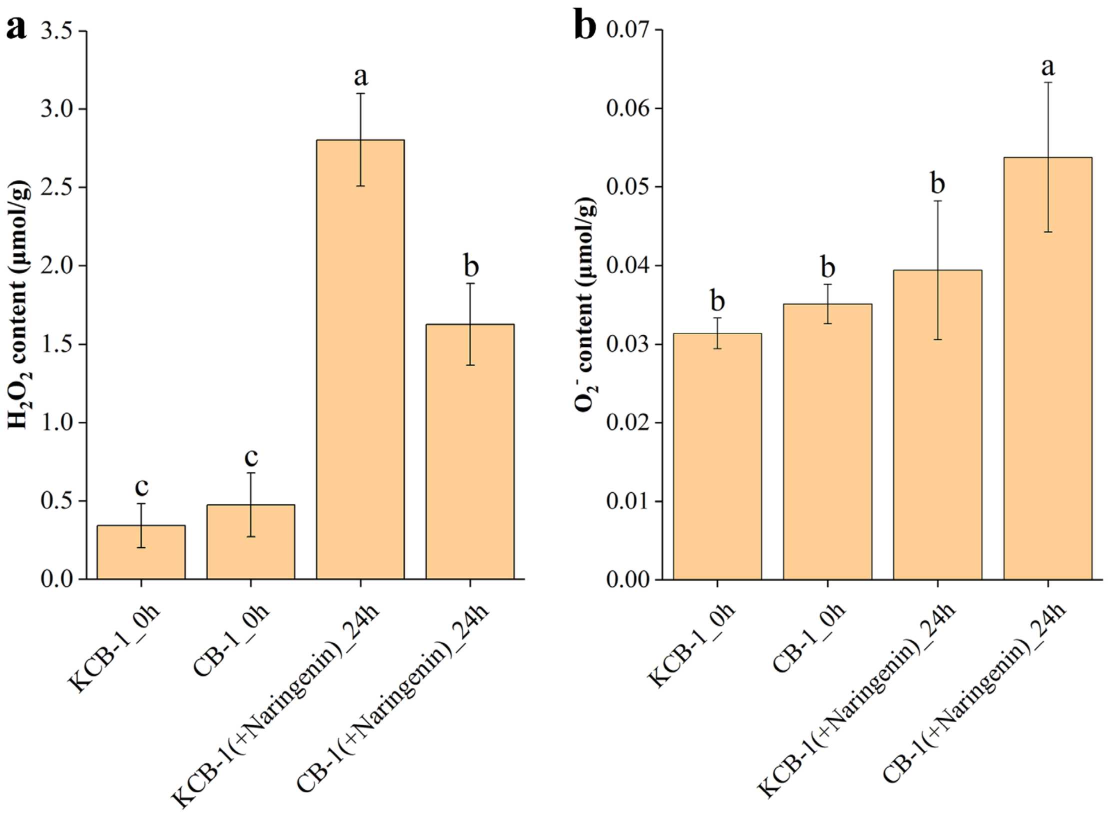

Supplemental Figure S10 Changes in the content of H_2O_2 and O^{2-} in roots after naringenin treatment of KCB-1 and CB-1. a. Changes in the content of H_2O_2 in roots after naringenin treatment of KCB-1 and CB-1. b. Changes in the content of O^{2-} in roots after naringenin treatment of KCB-1 and CB-1. Notes: Error bars represent Standard Deviation (SD). Between letters represent significant differences (One-way Anova, $p < 0.05$).

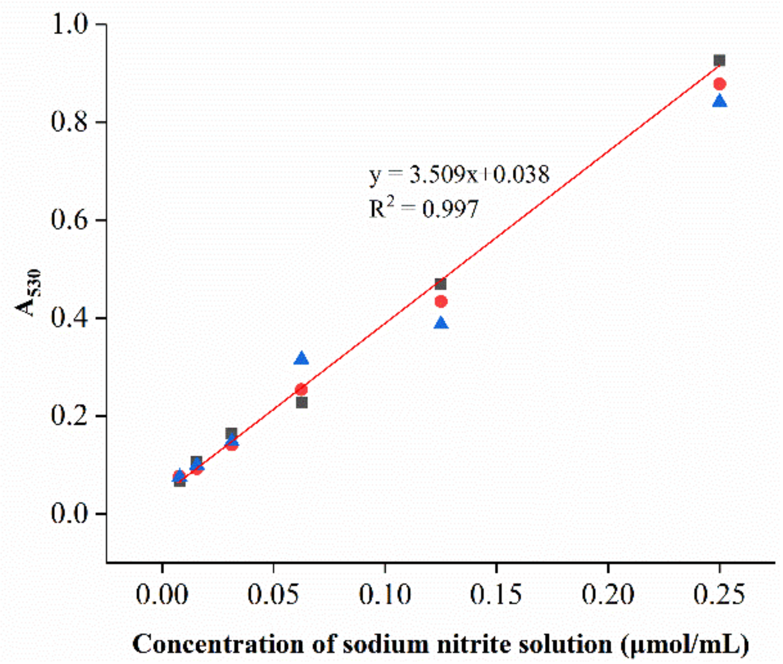

Supplemental Figure S11 Standard sample calibration curve for sodium nitrite solution.

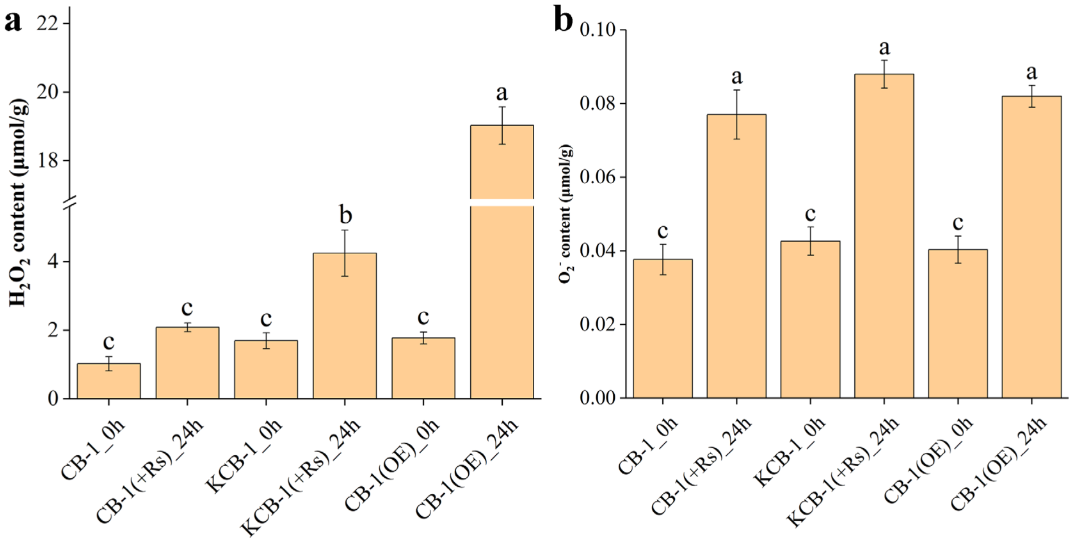

Supplemental Figure S12 Changes in the content of H_2O_2 and O^{2-} in roots after *R. solanacearum* treatment of KCB-1, CB-1 and CB-1(OE). a. Changes in H_2O_2 content in roots after *R. solanacearum* treatment of KCB-1, CB-1 and CB-1(OE). b. Changes in the content of O^{2-} in roots after *R. solanacearum* treatment of KCB-1, CB-1 and CB-1(OE). Notes: Error bars represent Standard Deviation (SD). Between letters represent significant differences (One-way Anova, $p < 0.05$).

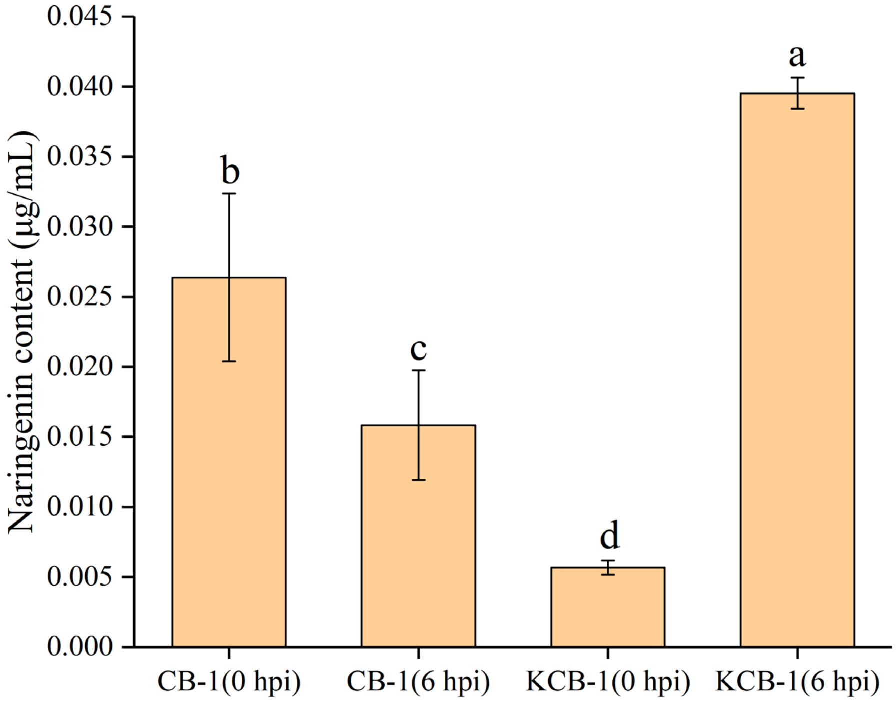

Supplemental Figure S13 Naringenin content in root secretions of KCB-1 and CB-1 at 0 and 6 hpi of inoculation with *R. solanacearum*. Letters represent significant differences between them. Notes: Error bars represent Standard Deviation (SD). Between letters represent significant differences (One-way

Anova, $p < 0.05$).

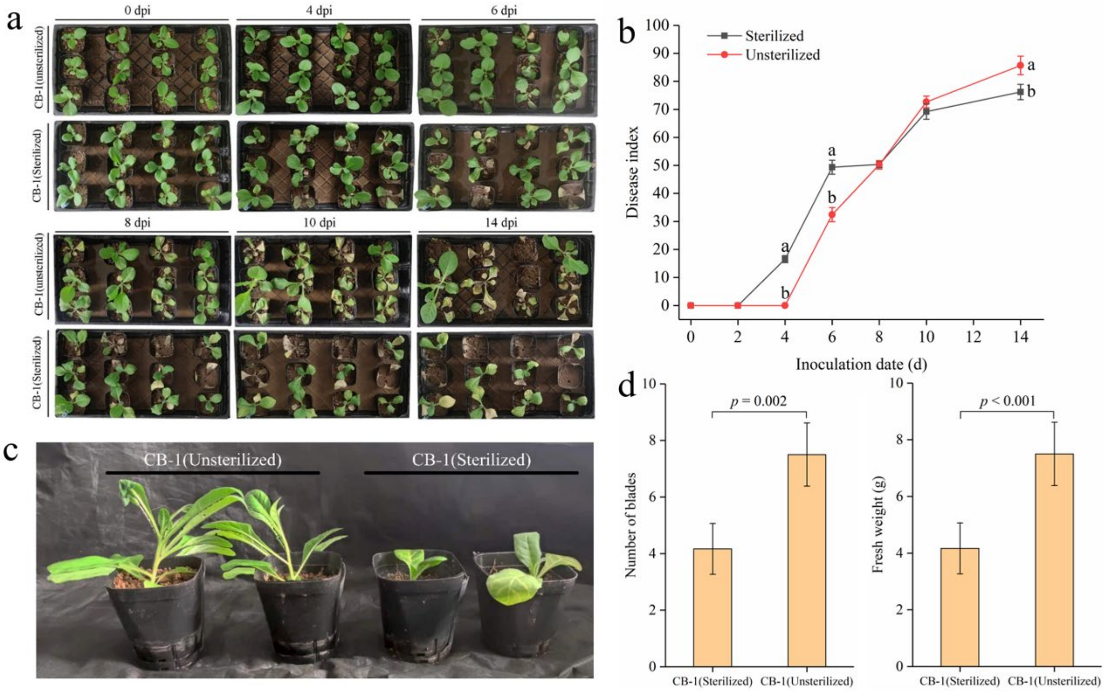

Supplemental Figure S14 Effect of sterilized and non-sterilized soil on CB-1 incidence and growth. a. CB-1 incidence in sterilized and non-sterilized soil after infestation with *R. solanacearum*. b. Statistics of CB-1 incidence index in sterilized and non-sterilized soil after infestation with *R. solanacearum*. c.CB-1 growth in sterilized and non-sterilized soil. d. Number of leaves and fresh weight of CB-1 in sterilized and non-sterilized soil. Letters between represent significant differences. Notes: Error bars represent Standard Deviation (SD). Between letters represent significant differences (Student's t test, $p < 0.05$).

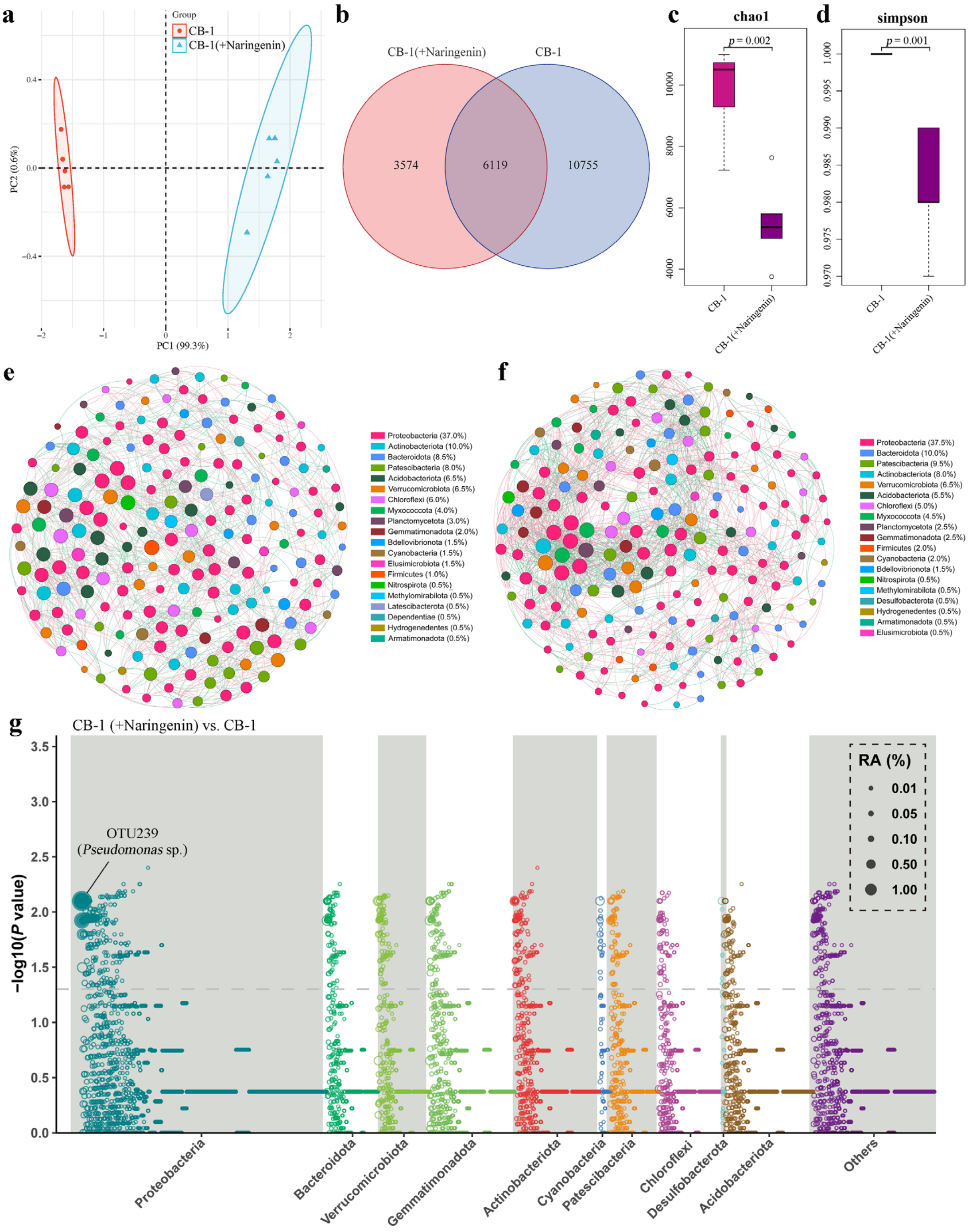

Supplemental Figure S15 Effect of naringenin on tobacco inter-root bacterial communities. a. PCA analysis of CB-1 and CB-1(+Naringenin) inter-root bacterial communities. b. Venn diagram of the number of shared and exclusive OTUs of CB-1 and CB-1(+Naringenin) inter-root bacterial communities. c. Chao1 analysis plot of CB-1 and CB-1(+Naringenin) inter-root bacterial communities.d. Simpson analysis plot of CB-1 and CB-1(+Naringenin) inter-root bacterial communities. e. Network plot of the top 20 CB-1 inter-root bacterial communities in abundance. f. Network diagram of CB-1(+Naringenin) inter-root bacterial communities in the top 20 of abundance. g. Manhattan plot of OTUs enriched in CB-

1 (+Naringenin) inter-roots relative to CB-1. The dashed line indicates the FDR-adjusted *p*-value significance threshold (p = 0.05). RA represents the relative abundance of OTU. Notes: Principal component (PC). Relative abundance (RA). The statistical method used in c-d. is Student's t test.

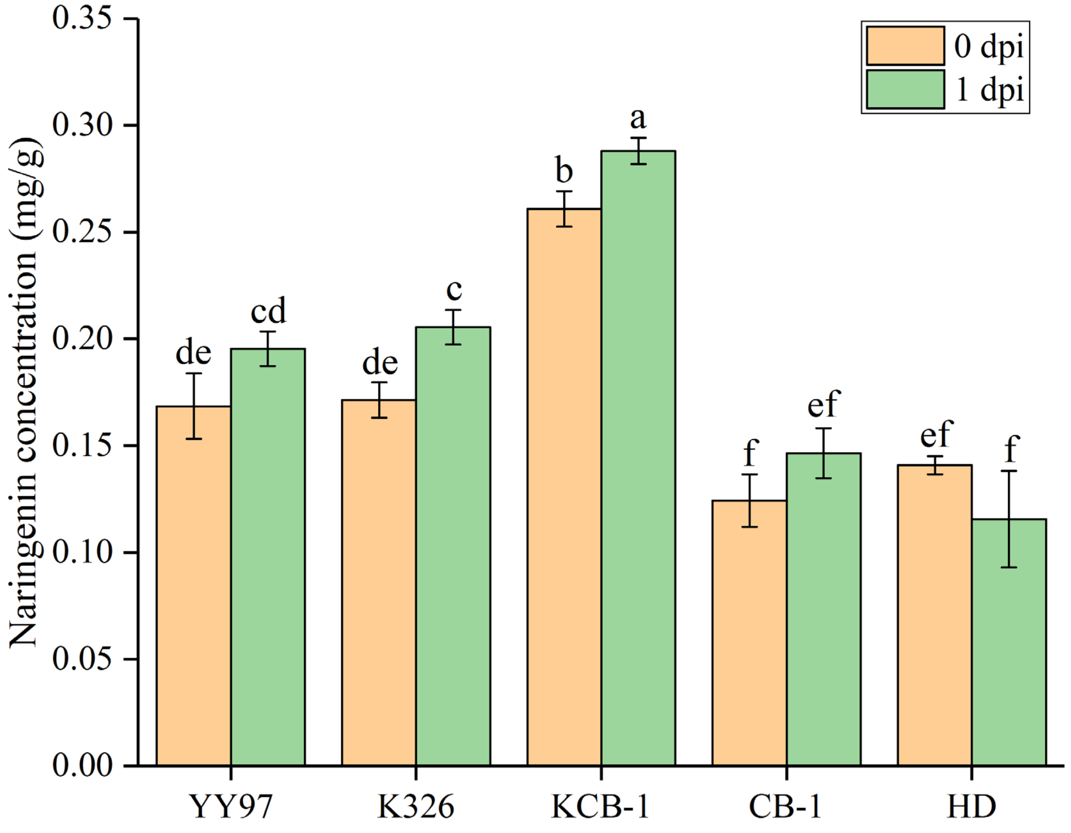

Supplemental Figure S16 Changes in naringenin content in roots of CB-1, KCB-1, HD, K326 and YY97 before and after inoculation with *R. solanacearum*. Notes: Error bars represent Standard Deviation (SD). Between letters represent significant differences (One-way Anova, $p < 0.05$).

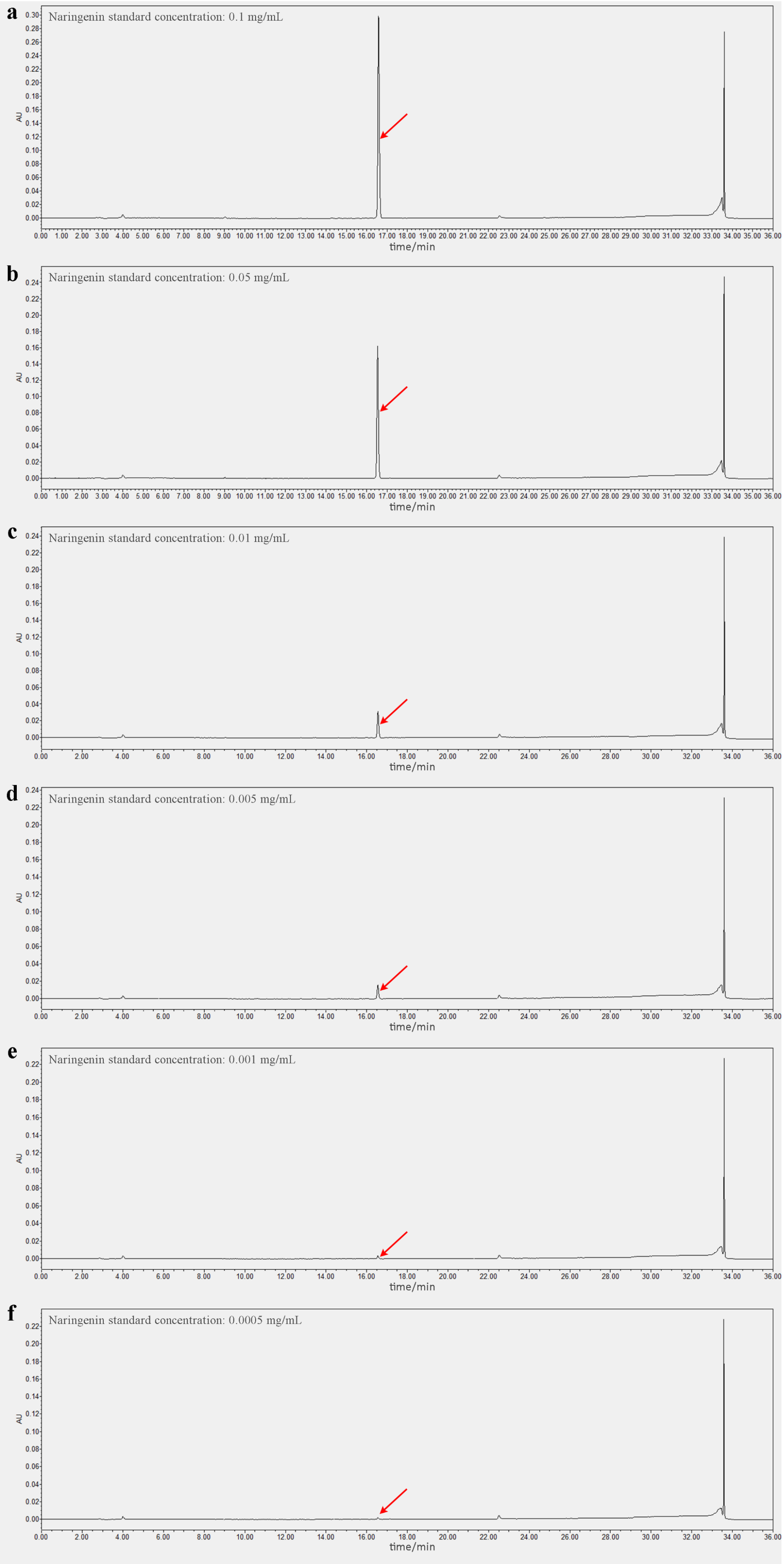
a
Naringenin standard concentration: 0.1 mg/mL
AU
time/min
b
Naringenin standard concentration: 0.05 mg/mL
AU
time/min
c
Naringenin standard concentration: 0.01 mg/mL
AU
time/min
d
Naringenin standard concentration: 0.005 mg/mL
AU
time/min
e
Naringenin standard concentration: 0.001 mg/mL
AU
time/min
f
Naringenin standard concentration: 0.0005 mg/mL
AU
time/min

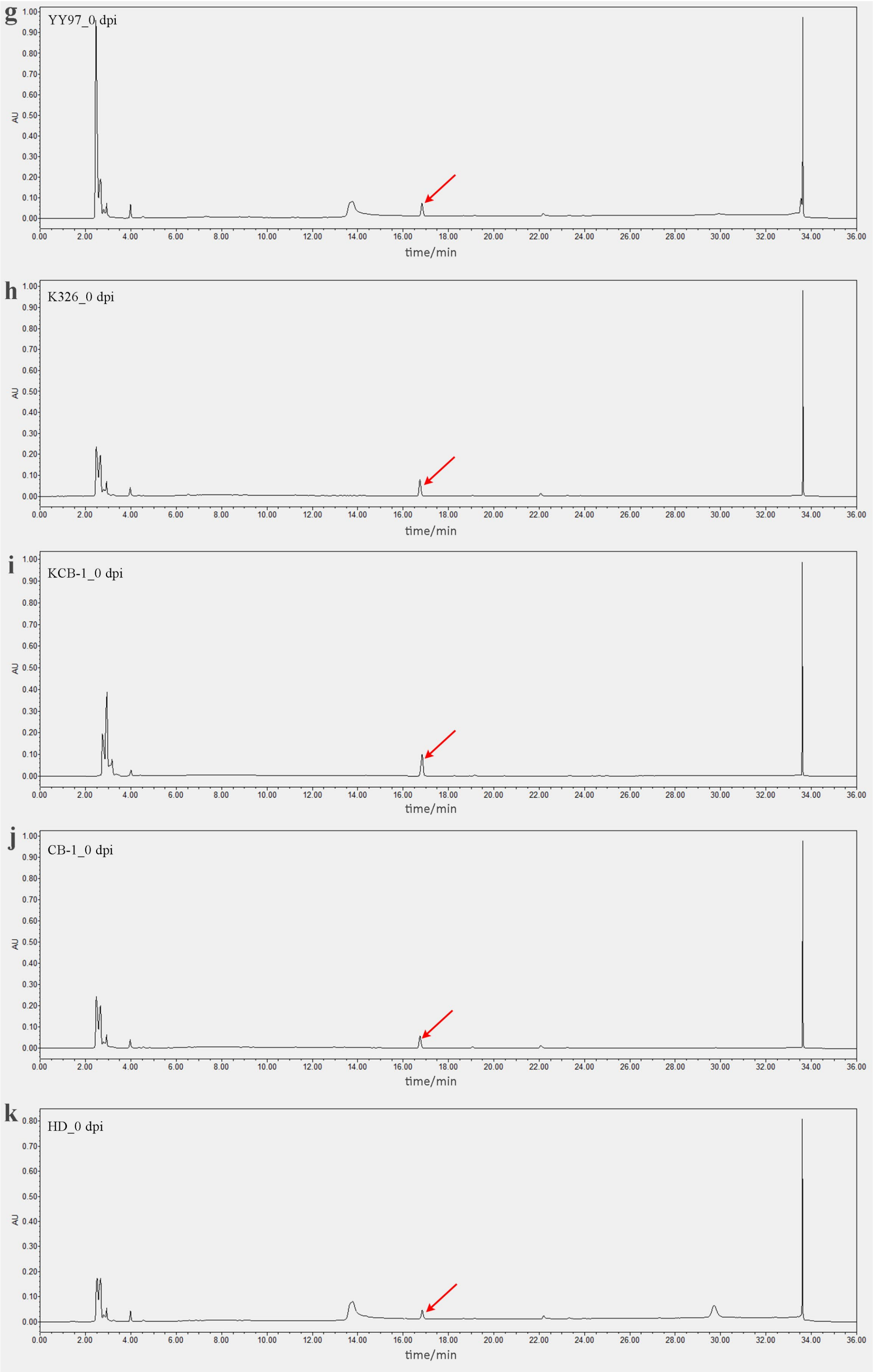
g
YY97_0 dpi
AU
time/min
h
K326_0 dpi
AU
time/min
i
KCB-1_0 dpi
AU
time/min
j
CB-1_0 dpi
AU
time/min
k
HD_0 dpi
AU
time/min
0.00 2.00 4.00 6.00 8.00 10.00 12.00 14.00 16.00 18.00 20.00 22.00 24.00 26.00 28.00 30.00 32.00 34.00 36.00

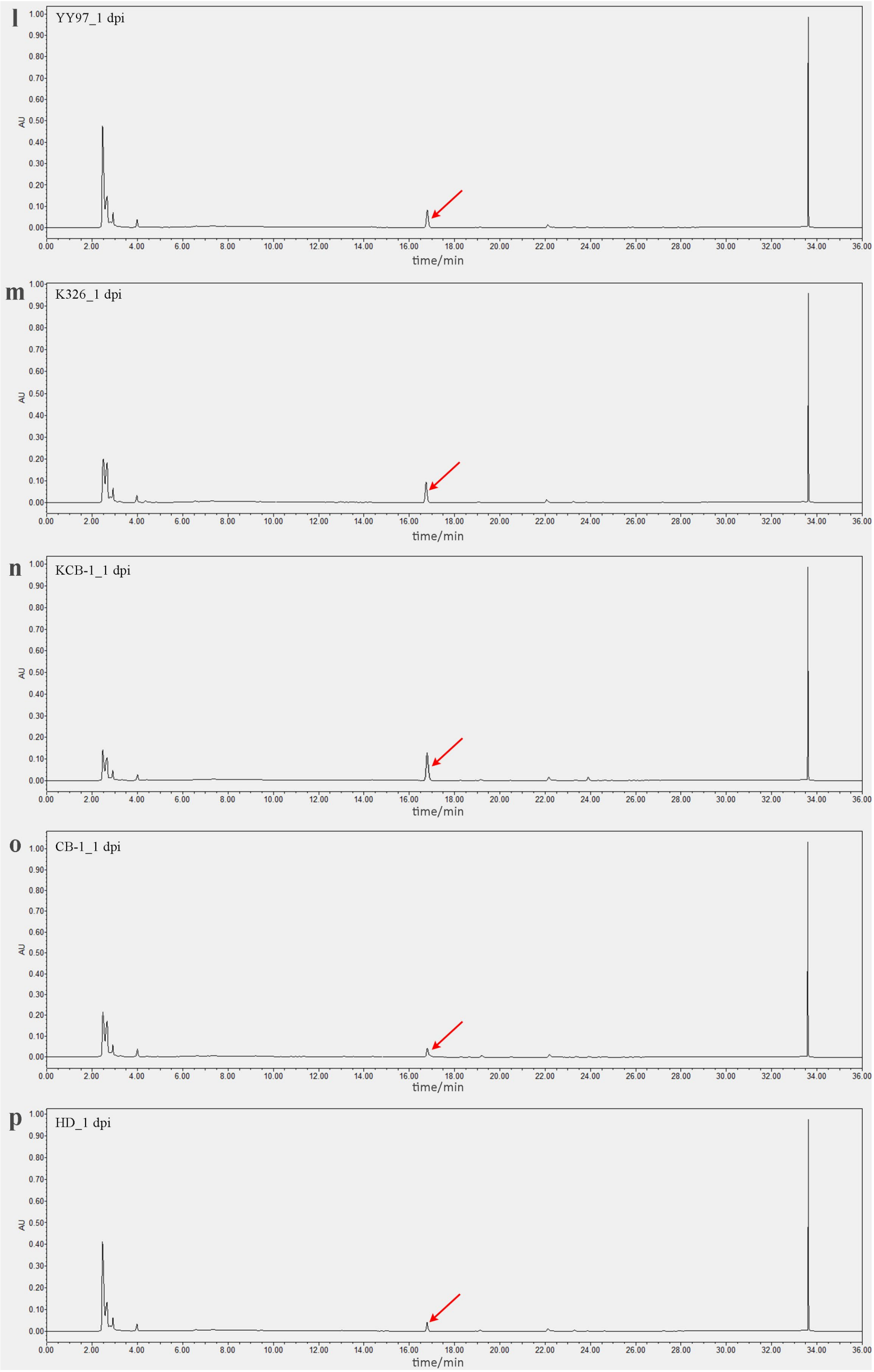

Supplemental Figure S17 HPLC assay of naringenin in tobacco roots. a-f. HPLC curves of naringenin standards at concentrations of 0.1, 0.05, 0.01, 0.005, 0.001 and 0.0005 mg/mL. g-k. HPLC curves of

naringenin content in roots of YY97, K326, KCB-1, CB-1 and HD at 0 dpi. l-p. HPLC curves of root naringenin content of YY97, K326, KCB-1, CB-1 and HD at 1 dpi. l-p. HPLC curves of root naringenin content of YY97, K326, KCB-1, CB-1 and HD at 1 dpi.

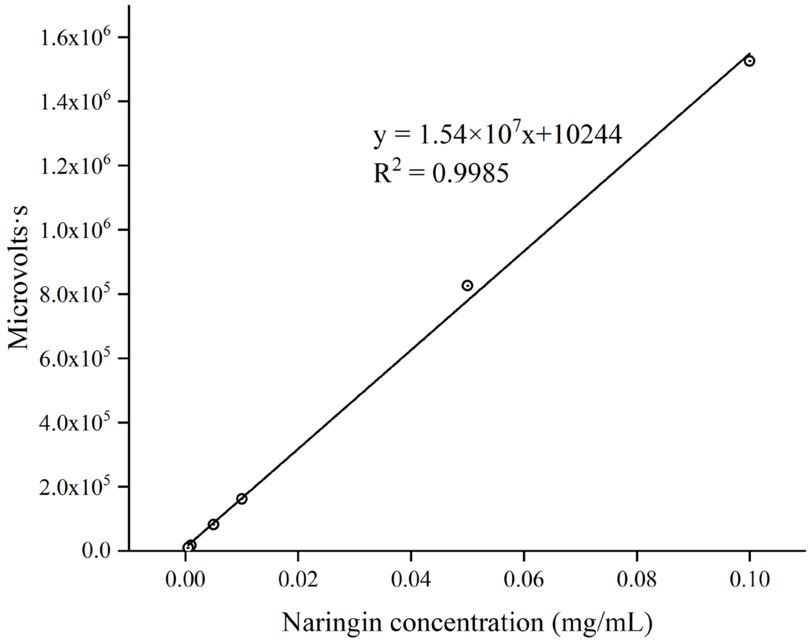

Supplemental Figure S18 Standard sample calibration curve for naringenin.

Supplemental Table S1. Minimum inhibitory concentration and minimum lethal concentration of naringin on *R. solanacearum*.

Colony name	Minimum inhibitory concentration (mg/L)	Minimum lethal concentration (mg/L)
Rs10-GFP2	200	300
RsFJ	300	300
Rs5	300	300
Rs7	300	400
Rs9	300	400
Rs11	200	300

Supplemental Table S2. Sequence information of the *egl* gene of *R. solanacearum*.

Number	Strain	Host	Origin	Accession number
1	R288	Mulberry	China	GQ907153*
2	JT523	Potato	Reunion Island	AF295252*

3	PSS81	Tomato	China	FJ561066*
4	PSS358	Tomato	China	EU407298*
5	UW151	Ginger	Australia	AF295254*
6	P11	Peanut	China	FJ561068*
7	GMI1000	Tomato	France	AF295251*
8	JT519	Geranium	Reunion Island	GU295032*
9	PSS219	Tomato	China	FJ561167*
10	O3	Olive Tree	China	FJ561069*
11	CIP365	Tomato	Philippines	GQ907151*
12	MAD17	Chili Pepper	Madagascar	GU295040*
13	GMI8254	Tomato	Indonesia	GU295014*
14	M2	Mulberry	China	FJ561067*
15	HBJS1	Tobacco	China	KP967641*
16	CFBP2972	Potato	Martinique	EF371809*
17	UW551	Geranium	Kenya	DQ657596*
18	JT525	Geranium	Reunion Island	AF295272*
19	MAFF301558	Potato	Japan	AY465002*
20	Psi7	Tomato	Indonesia	EF371804*
21	Y45	Tobacco	China	**
22	Rs10-GFP2	Tobacco	China	**
23	RSFJ	Tobacco	China	**
24	Rs5	Tobacco	China	**
25	Rs7	Tobacco	China	**
26	Rs9	Tobacco	China	**
27	Rs11	Tobacco	China	**
28	1609	Banana	South caribbean, Uraba, Colombia	RSIPO_02945***
29	BDBR229	Banana	Indonesia	BDB_120007***
30	CFBP2957	Tomato	Martinique	RCFBP_10201***

			(French West Indies)	
31	CMR15	Tomato	Cameroon	CMR15v4_mp10129***
32	PSI07	Tomato	Indonesia	RPSI07_mp0109***
33	Po82	Potato	Mexico	RSPO_m00159***
34	R24	Clove	Indonesia	RALSY_mp30777***

Note: *: Login number from NCBI; **: obtained by sequencing for this article; ***: Login number from LIPME Bioinformatics Platform (http://lipm-bioinfo.toulouse.inrae.fr/).

Supplemental Table S3. Primer sequences covered in the article.

**Primer name**	**Primer sequence**	**Reference**
1300-Grna-R	TATGTTCAATTTGTGAAATATCCCGG	This paper
PMV-U6sg_F	AATAGGCGTATCACGAGGCC	
PMV-U6sg_R	GCGAGTCAGTGAGCGAGGAA	
NtCHI_qPCR_F	ATTACGTGTTCCCATCAAC	
NtCHI_qPCR_R	CTTCACAAACTTCCCTTCA	
HygR336_F	AAGCCTGAACTCACCGCGAC	
HygR336_R	CGGTTGTAGAACAGCGGGCA	
pCAMBia35S_F	GACGCACAATCCCACTATCC	
pCAMBia35S_R	CATGGTGAGCAAGGGCGAGG	
35S: NtCHI1_F	acgaacgatagccatggtaccaATGGAGTCCATTACCATTGAGAATT	
35S：NtCHI1_R	atgttaacaaggcctggtaccCTAGACTCCAATTTCTGGAATGGTAG	
Endo-F	ATGCATGCCGCTGGTCGCCGC	
Endo-R	GCGTTGCCCGGCACGAACACC	

Supplemental Table S4. 35 flavonoids in targeted metabolomic assays.

Number	Name	Number	Name	Number	Name
1	Catechin	13	Silybin	25	Genistin
2	Genistein	14	Quercetin 3-glucoside	26	Glycitein
3	Puerarin	15	Fisetin	27	Glycitin

4	Baicalin	16	isovitexin	28	Liquiritigenin
5	Diosmin	17	Cynaroside	29	Quercitrin
6	Kaempferol	18	Apigenin	30	Vitexin
7	Luteolin	19	Astragalin	31	Dihydromyricetin
8	Rutin	20	Biochanin A	32	Naringenin
9	Daidzein	21	Daidzin	33	Quercetin
10	Naringin	22	Taxifolin	34	Kaempferide
11	lcariin	23	L-Epicatechin	35	Myricetin
12	Chrysin	24	Formononetin		

Supplemental method S1

The root tissues were removed from the fixative in a fume hood to trim the tissues of the target site with a scalpel, and the trimmed tissues were placed in a dehydration box. After that, the dehydration box was put into the dehydrator for sequential gradient alcohol dehydration. The root tissues were soaked in 75% alcohol for 4 h, 85% alcohol for 2 h, 90% alcohol for 2 h, 95% alcohol for 1 h, anhydrous ethanol for two times, each time for 30 min, benzene for 6 min, and xylene for two times, each time for 6 min, respectively. The wax-impregnated root tissues were then embedded in an embedding machine. The melted wax was placed in the embedding frame, and the tissue was removed from the dehydrating box and placed in the embedding frame before the wax solidified. The wax is then cooled on the freezer at -20 °C. After the wax has solidified, the wax blocks are removed from the embedding frame and the wax blocks are trimmed. The trimmed blocks were cooled at -20 °C on the freezer table, and then the cooled blocks were sliced in a paraffin slicer (Leica, Germany) to a thickness of 4 μm. The slices were floated on a slide spreader at 40 °C to flatten the tissues, and the slides were fished out of the tissues, and then slices were baked in an oven at 60 °C. The slices were dried in water and baked in wax, and then removed from the box. The slices were baked in an oven at 60 °C. After water-drying and wax baking, the slices were removed from the oven and placed sequentially into dewaxing clear solution I for 20 min, environmentally friendly dewaxing clear solution II for 20 min, anhydrous ethanol I for 5 min, anhydrous ethanol II for 5 min, and 75% alcohol for 5 min, and then washed with tap water. To show the cell walls of the roots, they were stained with Senka red and solid green. Sections were stained by placing them

into the fuchsin staining solution for 2 h and washed with tap water for 30 s to wash away the excess dye. Sections were placed in 50%, 70% and 80% gradient alcohol for 5 s. After that, sections were stained in plant solid green staining solution for 20 s and dehydrated in anhydrous ethanol. After that, the sections were put into clean xylene for 5 min, and neutral gum was used to seal the sections. Microscopic examination and image acquisition were performed under a microscope.

Supplemental method S2

The medium was gently rinsed with 1 × PBS (Servicebio, Wuhan), and then 1 × PBS was discarded and electron microscope fixative (Servicebio, Wuhan) was added. The samples were fixed at room temperature for 2 h and then transferred to 4 °C for storage. The fixed samples were rinsed three times with 0.1 M phosphate buffer (pH = 7.4) for 15 min each time. 0.1 M phosphate buffer (pH = 7.4) with 1% osmiic acid was fixed at room temperature and protected from light for 1-2 h. 0.1 M phosphate buffer (pH = 7.4) was rinsed three times with 15 min each time, and then the tissues were sequentially put into 30%-50%-70%-80%-90%-95%-100%-100%. 95%-100%-100% alcohol for 15 min each time and isoamyl acetate (China National Pharmaceutical Group Chemical Reagent Co., Ltd, China) for 15 min. The samples were then dried in a critical point dryer (Quorum, UK). The samples were placed on the conductive carbon film double-sided tape and placed on the sample stage of an ion sputtering apparatus (HITACHI, Japan) for about 30 s for gold spraying. Afterwards, the pickup image was observed with a scanning electron microscope (HITACHI, Japan).

Naringenin treated and untreated *R. solanacearum* were collected by centrifugation, discarded from the culture medium and fixed with electron microscope fixative for 2 h at room temperature, and then transferred to 4 °C for storage. Afterwards, the cultures were centrifuged, and the supernatant was added with 0.1 M phosphate buffer (pH = 7.4), mixed and rinsed for 3 min, then centrifuged, and the washing was repeated three times. The heated 1% agarose solution was cooled and added to a 1.5 mL EP tube, and the precipitate was picked up with tweezers and suspended in agarose before the agarose solidified. Tissues were then fixed in 1% osmium acid (Ted Pella Inc, USA) prepared in 0.1 M phosphate buffer (pH = 7.4) for 2 h. Tissues were rinsed three times in 0.1 M phosphate buffer (pH = 7.4) for 15 min each time, and then dehydrated sequentially in 30%-50%-70%-80%-95%-100%-100% alcohol for 20 min each time. Tissues were dehydrated in 30%-50%-70%-80%-95%-100%-100% alcohol for 20 min each time, and 100% acetone (China National Pharmaceutical Group Chemical Reagent Co., Ltd, China) for 15 min each time for two times, followed by incubation in acetone : 812 embedding agent (SPI, China)

= 1 ∶ 1 for 2-4 h at 37 °C, acetone : 812 embedding agent = 1 ∶ 2 for 2-4 h, and acetone : 812 embedding agent = 1 ∶ 2 for 2-4 h. Pure 812 embedding agent was poured into the embedding plate and the samples were inserted into the plate and baked in an oven at 37 °C for 5-8 h. The plate was placed at 60 °C for 15 min each time. The plates were polymerized in an oven at 60 °C for 48 h. The resin blocks were removed and set aside. The resin blocks were sliced at 60-80 nm in an ultrathin slicer, and sliced with a 150-mesh copper mesh. The copper mesh was stained with 2% uranyl acetate saturated alcohol solution for 8 min, washed with 70% alcohol for 3 times, washed with ultrapure water for 3 times, stained with 2.6% lead citrate solution for 8 min, washed with ultrapure water for 3 times, and then dried with filter paper. The copper mesh sections were put into the copper mesh box and dried at room temperature overnight. After treatment, the slices were observed under a transmission electron microscope (Hitachi, Japan), and the images were collected and analyzed.

Supplemental method S3

100 µL of *R. solanacearum* suspension (OD_{600} = 0.1) was added to 50 mL of NB culture medium containing naringenin, respectively. The final concentrations of naringenin were 100, 200, 300 and 400 mg/L. The control was treated with 100 µL of NB culture solution. The 50 mL sterile tubes to which *R. solanacearum* suspension was added were incubated at 28 °C for 24 h. Optical density (OD) was measured at 600 nm with a visible spectrophotometer at 6 h, 12 h and 24 h. The results of the incubation were summarized in the following table. Three replicates were made for each treatment.

Supplemental method S4

Swimming motility of *R. solanacearum* was assayed in semi-solid medium as described in a previous study (Tans-Kersten *et al.*, 2004). Briefly, naringenin was added to semi-solid medium to 100, 200, 300 and 400 mg/L. 2 µL of *R. solanacearum* suspension (OD_{600} = 0.5) was added dropwise to the plates. Inoculated plates were incubated in an incubator at 28 °C. The diameter of the surrounding white area was measured at 24 and 48 h of inoculation in three replicates each.

Supplemental method S5

20 mL of naringenin (300 mg/L) was poured over the roots of 2-month-old CB-1. The same volume of water was used as a negative soil dip test. The same volume of water was used as a negative control. After 24 h of watering, 10 mL of *R. solanacearum* suspension (OD_{600} = 1) was poured into the soil to inoculate single tobacco plants. The inoculated tobacco was placed in a greenhouse at 28 °C. The light/dark cycle was 12/12 h. Symptoms of each plant were scored using a 0-4 disease index. (0: no

symptoms present; 1: 25% of leaves wilted; 2: 26-50% of leaves wilted; 3: 51-75% of leaves wilted; 4: 76-100% of leaves wilted). A single treatment for each independent trial contained 10 plants and the assay was repeated three times. DI and control efficiency (CE) were determined using the following equation (Han *et al*., 2021b):

$$DI = (\sum(ni\times vi))/(N\times 4) \times 100$$

ni = the number of plants with the respective disease index, vi = DI (0, 1, 2, 3, and 4), and N = the total number of plants used in each treatment.

$$CE = (CK - T)/CK \times 100\%$$

T = the disease index of treatment, and CK = the disease index of the control group.

Supplemental method S6

DNA of R. solanacearum Rs10-GFP2, RS_FJ, Rs5, Rs7, Rs9, Rs11 was extracted by using Bacterial Genomic DNA Extraction Kit (TIANGEN, China), and the egl gene fragment of R. solanacearum was amplified with primers Endo-F and Endo-R, and the PCR products were sequenced after detection by 1% agarose gel electrophoresis. The remaining R. solanacearum variabilis egl gene sequences were obtained from LIPME Bioinformatics Platform (http://lipm-bioinfo.toulouse.inrae.fr/) and NCBI. The egl sequences of the 34 R. solanacearum variants were aligned and a phylogenetic tree was constructed using MEGA.

Supplemental method S7

The *CHI* sequences of CB-1 and KCB-1 were amplified with primers, the *CHI* sequence of K326 was obtained from the China Tobacco Genome Database (http://218.28.140.17/), and the *CHI* sequences of the rest of the species were obtained from NCBI. phylogenetic trees were constructed with MEGA.

Supplemental method S8

To generate the 35S::*NtCHI* vector, *NtCHI* was amplified from the root cDNA of CB-1 using primers, respectively. the cloning vector puc57 was digested with *Pvu*II , and the amplified product was later cloned into the puc57 cloning vector. The product was then cloned into the puc57 cloning vector and transformed into TOP10 for propagation. The plasmid of the positive strain was extracted as a template, and the *NtCHI* gene was amplified using 35S:*NtCHI*_F and 35S:*NtCHI*_R. The pCAMBia35S-EGFP vector was digested with *Kpn*I and *Bam*HI, while the amplified product was ligated into the pCAMBia35S-EGFP linear vector using a homologous recombination kit. The constructed overexpression vector was transformed into agrobacterium rhizogenes strain (LBA4404), whereas

exosomes were prepared, selected and regenerated. Transformants were selected in thaumatin-containing medium. The primers HygR336_F and HygR336_R were used for initial validation of the selected plants. And then the expression of *NtCHI* was verified by qPCR.

To obtain *NtCHI1*-CRISPR knockout mutants, sgRNAs targeting 5'-CCTGGTTCTACCAATACTTT-3' (gRNA_F2: 5'-gattAAAGTATTGGTAGAACCAGG-3', gRNA_R2: 5'-aaacCCTGGTTCTACCAATACTTT-3') were ligated into the pMV-U6sg -*Bsa*I vector, while the entire AtU6 region of the pMV-U6sg - *Bsa*I vector was later cloned. The AtU6 region was subsequently ligated into the 1300-18bc-UBQ-Cas9A50 vector. The constructed CRISPR/Cas9 binary vector was transformed into agrobacterium rhizogenes strain (LBA4404) for transformation of embryonic healing tissues of KCB-1. After resistance screening, leaf DNA of grown KCB-1 seedlings was extracted, amplified with primers *NtCHI*_330bp_F and *NtCHI*_330bp_R, and sequenced to verify whether the knockdown was successful.

Supplemental method S9

The collected roots of tobacco were frozen in liquid nitrogen for 5 min and then dried in a freeze dryer for 24 h. 0.2 g of dried roots was added to 1 mL of naringenin extract and then heated in a metal bath at 80 °C for 2 h. The samples were cooled down to room temperature and then centrifuged at 10,000 g for 10 min, and the supernatant was taken as the sample to be tested. Naringenin was extracted using a kit purchased from Solarbio (Beijing, China). The naringenin extracted by naringenin extraction kit had been identified and verified by mass spectrometry in the development of the technology method. 1.18 mL of trifluoroacetic acid was added to 500 mL of ultrapure water and mixed well to be mobile phase A. Chromatographically pure acetonitrile was used as mobile phase B. A C18 column (4.6 × 250 nm) was used with a column temperature of 30 °C, an injection volume of 10 μL, a flow rate of 0.8 mL/min, a wavelength of 270 nm, and an individual walking time of 30 min. Acetonitrile : mobile phase A = 20 : 80 The mobile phase in the ratio of acetonitrile : mobile phase A = 20 : 80 was used to equilibrate the column, and the spiking was started after the baseline was stabilized. Naringenin standards were diluted to 0.1, 0.05, 0.01, 0.005 and 0.001 mg/mL with methanol. The standard curve of naringenin was plotted with the concentration of the standard as the horizontal coordinate and the peak area as the vertical coordinate. Naringenin content (mg/g) = x × V extraction/W. V = volume of extract, 1.5 mL, W = sample mass, g.

Supplemental method S10

KCB-1 and CB-1 seedlings were cultured in hydroponic solution with addition of Hoagland's nutrient solution and after 1 month six KCB-1 and CB-1 plants were taken from each plant and the root system of KCB-1 and CB-1 was washed quickly with flowing 1 × PBS solution. Three KCB-1 and CB-1 plants were taken, and the root systems were immersed in 50 mL centrifuge tubes containing 40 mL of *R. solanacearum* solution (OD_{600} = 0.5), respectively. After 6 h of incubation at room temperature, the root secretion solution was immediately collected, freeze-dried into lyophilized powder, accurately weighed, and stored in 1.5 mL centrifuge tubes at -80 °C for targeted metabolism assay. Flavonoids in the root secretions of KCB-1 and CB-1 were analyzed for targeted metabolomics (Table S1). Briefly, 50 mL of collected root secretions were freeze-dried, dissolved in 1 mL of 80% methanol, and filtered through a 0.22 μm membrane (Merck, Darmstadt, Germany). Afterwards, the root secretions were subjected to qualitative and quantitative flavonoid assays.

Supplemental method S11

The kit is a double antibody one-step sandwich enzyme-linked immunosorbent assay (ELISA). To the coated microtiter wells pre-coated with salicylic acid (SA) antibody, the specimen, standard, and HRP-labeled detection antibody are sequentially added, incubated and washed thoroughly. The color is developed with the substrate TMB, which is converted to blue by catalysis of peroxidase and to final yellow by acid. The shade of color is positively correlated with the salicylic acid (SA) in the sample. The absorbance (OD value) was measured at 450 nm using an enzyme meter to calculate the sample concentration. The specific steps included: firstly, take out the desired plate from the aluminum foil pouch after equilibrating at room temperature for 20 min, and seal the remaining plate with a self-sealing bag and put it back to 4 °C. The standard and sample wells were set up, and 50 μL of different concentrations of standards were added to each standard well. 10 μL of the samples to be tested were added to the sample wells first, and then 40 μL of sample diluent was added to the sample wells; the blank wells were not added. In addition to the blank wells, add 100μL of horseradish peroxidase (HRP)-labeled detection antibody to each of the standard wells and sample wells, seal the reaction wells with a sealing film, incubate for 60min at 37 °C in a water bath or thermostat, discard the liquid, pat dry on blotting paper, fill each well with the washing solution, let it stand for 1min, shake off the washing solution, pat dry on the blotting paper, and so on, repeat the plate washing for 5 times (a washing machine can also be used to wash the plate). Repeat this process 5 times (a plate washer can also be used to wash the plate). Add 50 μL of substrate A and B to each well, incubate at 37 °C for 15 min, add 50 μL of termination solution

to each well, and measure the OD value of each well at 450 nm within 15 min. Finally, the linear regression curve of the standard was plotted with the concentration of the standard as the horizontal coordinate and the corresponding OD value as the vertical coordinate, and the concentration value of each sample was calculated according to the curve equation.

Supplemental method S12

Three copies of roots of KCB-1, CB-1 and CB-1 (OE) before and after treatment were taken respectively. The contents of H_2O_2 and O^{2-} were detected with H_2O_2 and O^{2-} detection kits (Beijing Solarbio Science & Technology Co., Ltd, Beijing).

The roots of KCB-1 and CB-1 were treated with naringenin solution (concentration of 300 mg/L, dissolved in 1 mL of anhydrous ethanol) for 1 d. The control was treated with the same volume of water + anhydrous ethanol. Three portions of roots of KCB-1 and CB-1 were taken before and after treatment, respectively. The contents of H_2O_2 and O^{2-} were detected by H_2O_2 and O^{2-} detection kits (Beijing Solarbio Science & Technology Co., Ltd, Beijing).

Supplemental method S13

The test samples consisted of 5 portions each of inter-root soil of CB-1, KCB-1, CB-1(OE), CB-1(+Naringenin), each portion was 3 g. The samples were collected in 2 mL sterile centrifuge tubes and stored frozen in a -80°C refrigerator to wait for preparation. DNA quality and concentration were checked using a Nanodrop 2000 (ThermoFisher Scientific, Inc., USA), and DNA samples were stored at -20 °C for subsequent sequencing. 20 °C for subsequent sequencing. Bacterial 16Sr RNA was amplified using universal primers 338F (5'-ACTCCTACGGGAGGCAGCAG-3') and 806R (5'-GGACTACHVGGGTWTCTAAT-3') V3-V4 region of the gene. A barcode sequence of 8 bp was added at the 5' end of each of the upstream and downstream primers to distinguish the different samples. The final synthesized universal primers with barcode sequences were amplified on an ABI 9700 PCR instrument (Applied Biosystems, Inc., USA). The size of the amplified target bands was detected by 1% agarose gel electrophoresis, and the PCR products were purified automatically using the Agencourt AMPure XP (Beckman Coulter, Inc., USA) Nucleic Acid Purification Kit. The PCR products were used for library construction using the NEB Next Ultra II DNA Library Prep Kit (New England Biolabs, Inc., USA). Finally, the constructed libraries were purified using Agencourt AMPure XP (Beckman Coulter, Inc., USA) Nucleic Acid Purification Kit. The concentration of the libraries was roughly checked using Nanodrop 2000 (ThermoFisher Scientific, Inc., USA), the fragment size of the libraries was detected

using Agilent 2100 Bioanalyzer (Agilent Technologies, Inc., USA), and the fragment size of the libraries was measured using ABI StepOnePlus Real-Time PCR System (Applied Biosystems, Inc., USA) was used to detect the fragment size of the libraries and ABI StepOnePlus Real-Time PCR System (ABI) was used to quantify the library concentration. The final libraries were sequenced on the Illumina Miseq/Nextseq 2000/Novaseq 6000 (Illumina, Inc., USA) platform.
